# Reactive Additive-Induced Joining of Apolar Thiol-Coated Gold Nanoparticle Cores at Low Temperatures

**DOI:** 10.1021/acs.langmuir.6c00514

**Published:** 2026-06-09

**Authors:** Tobias Valentin Knapp, Bart-Jan Niebuur, Olga Matsarskaia, Tobias Kraus

**Affiliations:** † 28391 INM—Leibniz Institute for New Materials, Campus D2 2, 66123 Saarbrücken, Germany; ‡ ILL—Institut Laue Langevin, 71 Avenue des Martyrs, 38042 Grenoble, France; § Saarland University, Colloid and Interface Chemistry, Campus D2 2, 66123 Saarbrücken, Germany

## Abstract

The agglomeration of apolar metallic nanoparticles (NPs) brings them within nanometer distances, enabling nonradiative coupling through electron conduction tunneling and plasmonic interactions. Even stronger coupling can be achieved with metallic connections between the particles. Thermally induced coarsening is often used to join particles in this way, but typically requires high temperatures. For example, the thermal coarsening of thiol-coated NPs in apolar dispersions requires heating above 120 °C to induce ligand desorption. This leads to side reactions, notably Ostwald Ripening and the formation of small particles. Here, we introduce a new method for controlled NP coarsening in apolar dispersions at near-ambient conditions. Small amounts of reactive chemical additives are added to the dispersion and thermally activated. Transmission electron microscopy and small-angle X-ray scattering reveal that adding the cyclic sulfide additive molecule tetrahydrothiophene (R5S) to a dispersion of hexadecanethiol (HDT)-coated gold nanoparticles (AuNPs) induces aggregation at temperatures as low as 40 to 60 °C. The cores form metallic joints, forming larger continuous gold bodies. An Arrhenius analysis of the temperature-dependent aggregation kinetics identifies the exchange of HDT to R5S on the gold surfaces as the rate-limiting step. Small-angle neutron scattering and thermogravimetry confirm the mechanism and indicate that ∼80% of HDT is replaced by R5S after 5 h at 50 °C. Progressive ligand exchange reduces the steric repulsion by the shells until aggregation sets in. A comparison with the larger cyclic sulfide thionane and the cyclic amine pyrrolidine, which do not induce coarsening, shows that the prerequisite for ligand desorption at moderate temperatures to enable AuNP coarsening is a small size and strong affinity of the additives to the gold surfaces.

## Graphical abstract

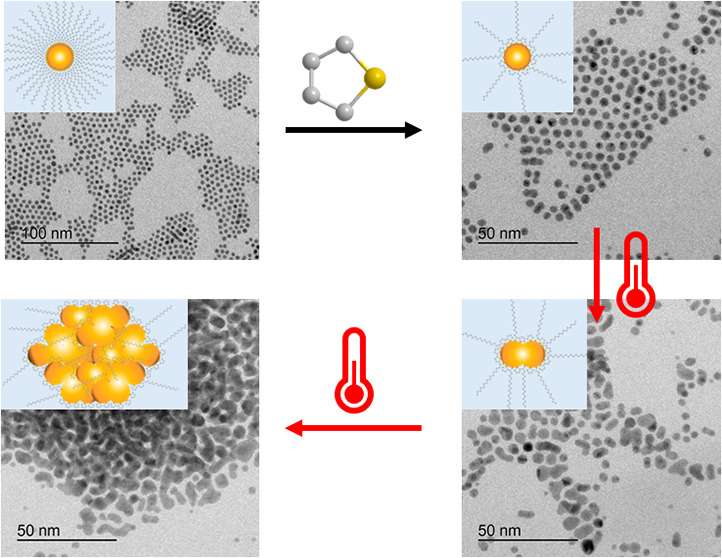

## Introduction

The assembly of apolar metallic nanoparticles in dispersions reduces interparticle distances to the nanometer scale, enabling strong coupling through nonradiative mechanisms such as electron conduction and tunneling,^1^ and strong plasmon resonance coupling.^2^ This has been exploited to create functional layers from gold nanoparticles (AuNPs) that are stable, easy to modify chemically, and have attractive optical and electronic properties.^3,4^ Such layers are suitable for sensing,^5,6^ catalysis,^7,8^ and printable electronics.^9–11^

Apolar AuNPs are typically stabilized by an organic ligand shell^12^ with amine,^13^ phosphine,^14^ or thiol functionalities^15^ that bind the nanoparticle surfaces. Previous studies have shown that apolar AuNP agglomeration upon cooling is affected by a disorder-to-order transition of the ligand shell and the attraction of cores.^16,17^ The ligand shell structure depends on the molecular structure of the ligands, which in turn affects their colloidal stability.^18–21^ The balance between core attraction, ligand shell structure, and ligand-solvent interactions limits interparticle coupling. Very small spacings would require very thin ligand shells that are unable to stabilize the dispersion.^17,22–25^

It is often useful to establish metallic connections between the particle cores. This can, for example, be exploited to remove electrical contact resistance between printed particles in electronics^11^ or to alter plasmonic absorption bands strongly.^26^ Metallic connections can also enhance the mechanical stability of nanoparticle assemblies, enabling the formation of porous gold-based structures from dispersions^27^ as well as complex ceramic–metallic bulk architectures.^28^ The most common route to metallic connections is the thermally induced coarsening of metal nanoparticles.

In dispersions, elevated temperatures—typically above 120 °C—have been used for the coarsening of apolar, thiol-coated AuNPs.^29,30^ Coarsening has been primarily attributed to the thermal desorption of ligands from the nanoparticle surface that reduces the stabilizing and repulsive effect of the ligand shell.^29,31,32^ As a result, reactions between different cores are possible. Surface diffusion of gold atoms allows Ostwald ripening after desorption of ligands. If the collision energy between AuNPs is sufficiently high, cores can coarsen by coalescence.^29,31^

The temperature required for ligand desorption depends strongly on the chemical nature of the binding group. For example, linear alkyl thiols typically desorb between 120 and 180 °C.^33^ Smaller^33^ or sterically more demanding thiols (that are branched in the α-position)^23^ have desorption temperatures that are 20 to 30 K lower than the desorption temperature of linear thiols, depending on the molecular structure.^34^ Linear alkyl amines desorb at even lower temperatures, typically between 50 and 120 °C.^35^ Coarsening in dry nanoparticle superlattices has been reported for significantly lower temperatures. For example, coalescence of oleylamine-coated gold nanoparticles with a diameter of 8.1 nm has been observed at 70 °C, facilitated by reduced ligand density at the (111) facets and strong interparticle attractions in the dry film that displace ligands from the interface.^36^

Coarsening has been reported for nanocomposites, too. Meli et al. demonstrated that self-assembled dodecanethiol-coated AuNPs embedded in a poly­(methyl methacrylate) matrix coarsened in two stages at 150 °C.^31^ During the first 480 min, coarsening proceeded via simultaneous Ostwald ripening and coalescence. This was initiated by ligand desorption, which reduced surface passivation and allowed the diffusion of atoms or small clusters, thereby enabling ripening. As particle sizes increased within the assembly, the interparticle spacing decreased. Once the gap between particle cores fell below approximately 0.5 nm, a second stage dominated by coalescence set in.

We are interested in reactive coarsening processes that join metal nanoparticle cores without side reactions at low temperatures. Such routes are interesting for the “chemical sintering” of printed nanoparticle films into electrically conductive leads,^37^ the transformation of plasmonic particles in sensing to elicit a strong optical response,^26,38^ and the formation of metal nanoparticle aerogels,^39^ for example. Few studies have explored the use of reactive additives that modify nanoparticle surfaces and lower the temperatures required for nanoparticle coarsening. In polar dispersions of AuNPs, Kim et al. reported coalescence induced in citrate-coated AuNPs by the addition of benzyl mercaptan at room temperature.^40,41^ Lee et al. studied the coarsening of citrate-covered AuNPs in aqueous solutions using thiolated poly­(ethylene glycol) as a destabilizing agent, also at room temperature.^27^ Both found that a partial ligand exchange of citrate on the gold surface reduced the zeta potential and the protective effect of the shell sufficiently to induce aggregation of the AuNPs. In dry films or nanoparticle assemblies, coarsening temperatures are usually above 150 °C.^32,42^ Halide-containing surfactants, like tetraoctylphosphonium bromide (TOPB) or tetraoctylammounium bromide (TOAB) lower the coalescence temperature of dodecanethiol-coated AuNPs in dry films from 190 to 205 °C without surfactants down to 165 to 175 °C.^43^ The oxidation of thiol ligands upon air exposure was found to decrease their binding affinity to the gold surface, reducing the shell stability and thereby increasing the reactivity of AuNPs in dry films.^44,45^ To our knowledge, no reports have addressed the reduction of coarsening temperatures for dispersed apolar gold nanoparticles. Enabling reactive coarsening at low temperatures to nonpolar systems would remove material constraints and significantly broaden its applicability.

Here, we show that small surface-reactive molecules can be used to join apolar gold nanoparticle cores at low temperature. Our previous work showed that the cyclic sulfide tetrahydrothiophene (R5S) partially replaces the initial hexadecanethiol (HDT) ligands on the AuNP surfaces.^46^ This ligand exchange lowered the order–disorder transition temperature of the shell, while the larger cyclic sulfide thionane (R9S) and cyclic amine pyrrolidine (R5N) did not significantly alter the ligand shell structure. Here, we report on the reduction of the AuNP coarsening temperatures in apolar dispersions using small molecular additives.

We used transmission electron microscopy (TEM) and small-angle X-ray scattering (SAXS) to analyze the coarsening of the initially spherical gold cores in the presence of R5s, R5N and R9S at temperatures up to 60 °C. The combination of thermogravimetric analysis (TGA) and small-angle neutron scattering (SANS) was used to detect changes in the ligand shell structure, induced by the exchange of the initial HDT ligand by the additives. We found that R5S enables the coarsening of initially HDT-stabilized AuNPs in *n*-decane by aggregation, while pyrrolidine (R5N) and thionane (R9S) did not show such an effect.

## Results and Discussion

Spherical nanoparticles (AuNPs) with core diameters of approximately 4 nm and hexadecanethiol (HDT) ligand shells (“AuNP” in the following) were synthesized using well-established methods^47,48^ and dispersed in *n*-decane at a gold concentration of 2.5 mg/mL. Tetrahydrothiophene (R5S), pyrrolidine (R5N), and thionane (R9S) were added as additives at various concentrations. The dispersions were stored for 24 h at room temperature after the addition of the additives.

We followed the subsequent coarsening of the gold cores at elevated temperatures of 40 °C, 50 °C, and 60 °C. Coarsening is the structural evolution of the primary AuNP dispersion toward a coarser structure and an increase of the characteristic size scale. We use it as an umbrella term mechanisms that include aggregation, (Ostwald) ripening and others. Aggregation is the process by which primary particles join and form larger structures, termed aggregates. Coarsening via Ostwald ripening, in contrast, requires atoms or small clusters that detach from one particle, diffuse through the surrounding medium, and redeposit onto another particle. This leads to the growth of larger particles at the expense of smaller ones. We used transmission electron microscopy (TEM) and in situ time-dependent small-angle X-ray scattering (SAXS) to investigate the coarsening kinetics and to distinguish coarsening mechanisms. TEM provided an impression of the AuNP structure in real space, while SAXS allowed to study their structural properties *in situ* with statistically relevant results. The ligand shell was characterized by small-angle neutron scattering (SANS), thermogravimetric analysis (TGA) and SAXS. SANS characterized the ligand shell thickness and composition during the initial stages, while TGA was used to determine the shell composition during all stages of AuNP coarsening. The results are discussed in the following.

### Stages of AuNP Coarsening in the Presence of R5S

A AuNP dispersion containing an R5S concentration of 128 mM was heated to 50 °C in a vial (1.5 mL). At selected times, aliquots were taken from the dispersion, cooled to room temperature, and analyzed using TEM. The obtained micrographs were analyzed using label analysis to determine the particle sizes and size distributions (see Materials and Methods section for details).

Figure 1 shows TEM images after 0, 150, and 300 min, illustrating a continuous change in core geometry. The original, additive-free particle dispersion was fully dispersed (see Figure S1a in the Supporting Information). At *t* = 0 min, primary AuNPs with an average diameter of *d* = 3.8 nm and a variance of σ = 0.25 (Figure 1d) dominated the dispersion (89%). Only 7% of the particles had *d* < 3.0 nm and 4% of the particles had *d* < 4.5 nm. The small fraction of large particles is composed of aggregates consisting of a few primary particles (Figure 1a), that formed during the 24 h storage time after the addition of R5S.

**Figure 1:**
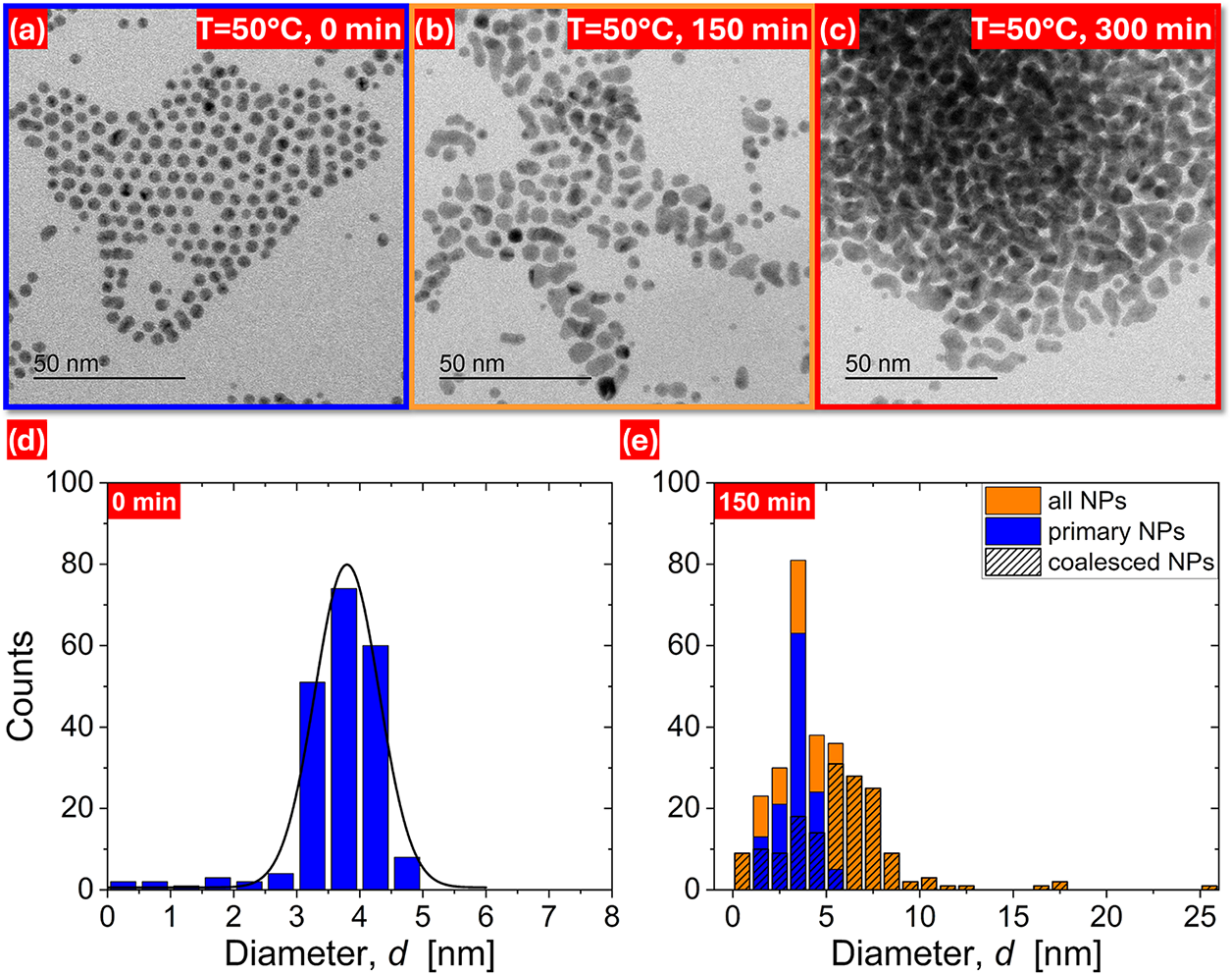
Coarsening of AuNPs coated with HDT and dispersed in *n*-decane at 50 °C in the presence of R5S at 128 mg/mL. Transmission electron micrographs show the original dispersion (a) before heating, (b) after 150 min at 50 °C and (c) 300 min at 50 °C. The particle size distribution determined from (a) is shown in (d). The particle size distribution after 150 min at 50 °C was determined using three TEM images (see Figure S3 in the Supporting Information) and is shown in (e). Blue bars in (d, e) represent spherical particles (circularity above 0.6), while black dashed bars represent nonspherical particles (circularity below 0.6). Orange bars show the combined size distribution.

After 150 min, aggregates formed (Figure 1b) that are clearly visible in the diameter distributions shown in Figure 1e. Analysis of their circularity with TEM (see the TEM section of the Supporting Information for a detailed description) suggests that 70% of the particles with a diameter between 1.5 and 4.5 nm were spherical, primary particles. Less than 5% of particles larger than 4.5 nm were spherical, suggesting that most were aggregates. The size distribution of primary particles was practically unchanged after 150 min, excluding Ostwald ripening as the main coarsening mechanism. Instead, we propose that particles aggregate and begin to fuse. This is consistent with reports indicating that temperatures >120 °C are required for diffusion processes between dispersed particles.^29,31^

After 300 min, large aggregates with diameters between 200–2000 nm were present (Figures 1c and S1b in the Supporting Information). These aggregates are the result of the continuous aggregation process during which both primary particles smaller aggregates join together and form larger structures in the μm-range. Figure 1c indicates that the smaller aggregates within these large assemblies were partly fused, but primary particles remained discernible. Densification due to complete coalescence was not observed during this stage.

### Mechanisms of AuNP Coarsening in the Presence of R5S

We used SAXS to determine the kinetics of aggregation as a function of temperature. Time-dependent scattering patterns were recorded at 50 °C in intervals of 10 min with an integration time of 5 min (Figure 2a,b).

**Figure 2:**
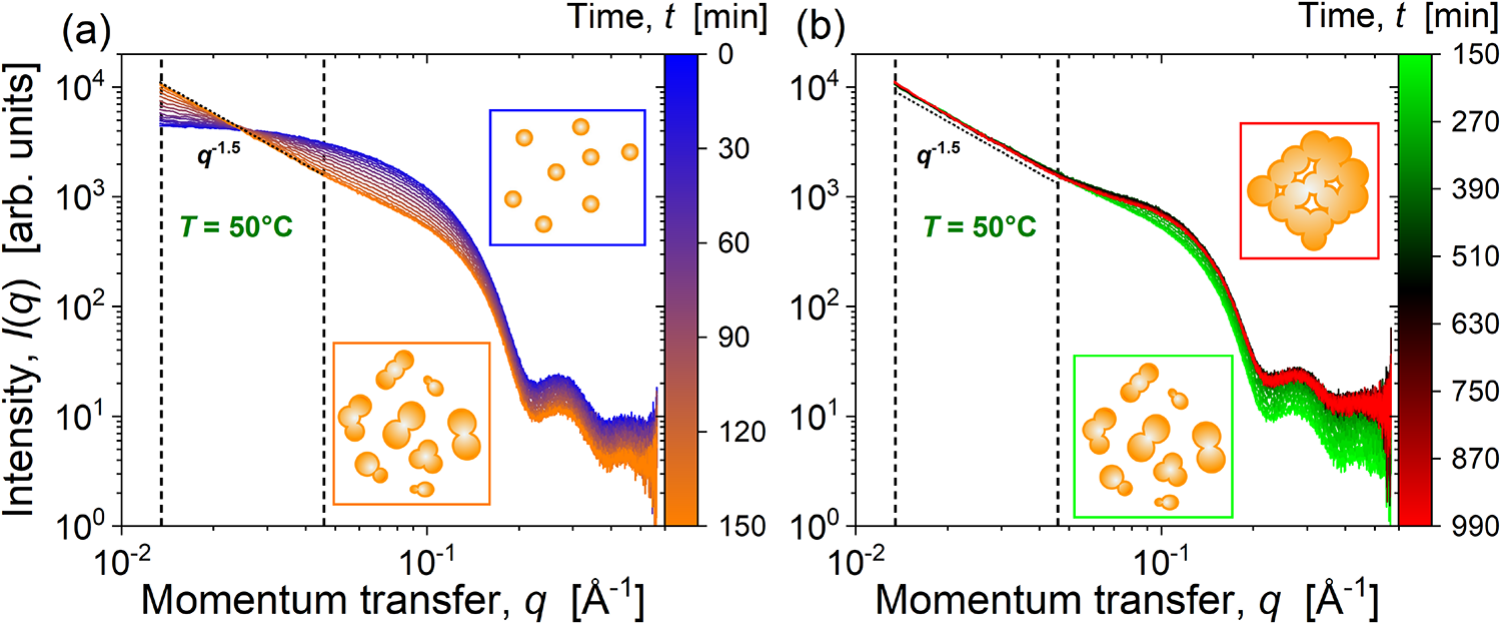
Time-dependent small-angle X-ray scattering patterns of AuNPs coated with HDT and dispersed in *n*-decane at 50 °C in the presence of R5S at 128 mg/mL. The data was divided into two graphs for clarity, with scattering patterns (a) between 0 and 150 min and (b) between 150 and 990 min.

The initial scattering from samples 24 h after mixing the AuNPs with R5S prior to heating (Figure 2a, blue curve) was characterized by an intensity plateau at 0.01 Å to 0.06 Å^–1^, followed by a decay in intensity at *q* > 0.06 Å^–1^ and a small peak at 0.27 Å^–1^. It was dominated by the form factor of spherical particles, indicating that no strong coarsening occurred at room temperature. During heating, the slope at low *q*-values (*q* < 0.04 Å^–1^) decreased during the first 150 min and then remained constant. The final slope of *q* ^–1.5^ indicates that the formed particles are mass fractals consisting of networks with a fractal dimension of 1.5 at length scales of ∼10–100 nm,^49^ pointing to aggregation by direct collisions and fusion of primary particles, in agreement with the TEM observations above.

Further insights into the early stage of coarsening were gained from a quantitative analysis of the obtained SAXS patterns. TEM suggested that spherical, primary nanoparticles and small aggregates consisting of 2 primary nanoparticles were present at early times (0 to 70 min). Their scattering patterns were modeled using the equation

1 $$I_{\mathrm{tot}}\left(q\right)=I_{\mathrm{Porod}}\left(q\right)+I_{\mathrm{pS}}\left(q\right)+I_{\mathrm{pDum}}\left(q\right)+I_{\mathrm{bkg}}$$

with *I*_pS_(*q*) a form factor of polydisperse spheres with a Gaussian size distribution and *I*_pDum_(*q*) a form factor of dumbbell-shaped particles, i.e., two primary particles with a Gaussian size distribution partly fused together. To account for scattering from larger structures, i.e., aggregates containing more than 2 primary nanoparticles, a Porod term, *I*_Porod_(*q*), was added. Lastly, *I*_bkg_ accounts for background scattering. Full equations and details regarding the analysis are given in the Supporting Information.

The initial scattering pattern (Figure 3a) could satisfactorily be modeled using contributions from spherical nanoparticles and dumbbell-shaped aggregates, while a model considering only polydisperse spheres did not match the data well. This indicates that some aggregation takes place already during the 24 h storage at room temperature after the addition of R5S. Approximately 40 vol % of all particles in the dispersion were dumbbell-shaped, while 60 vol % were spherical primary particles. A Porod term to describe larger structures was not required, suggesting that the fraction of larger aggregates was small. Note that the aggregate fraction from SAXS is above that from TEM analysis. This is likely due to the similarity between the form factors of polydisperse spheres and dumbbells and the statistical limitations of TEM analyis.

**Figure 3:**
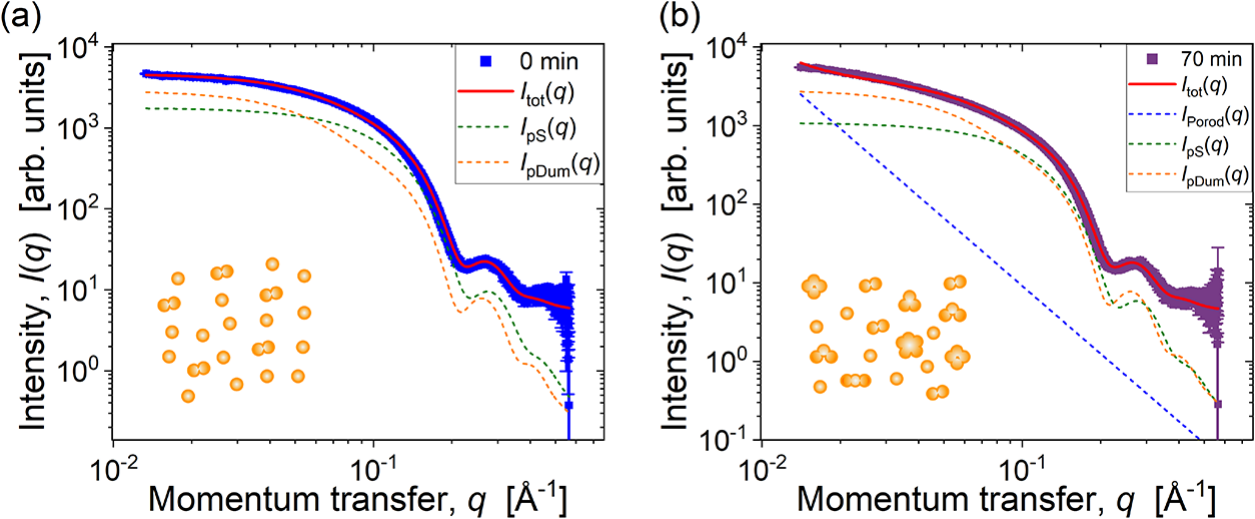
Model-based analysis of SAXS from coarsening AuNPs dispersions coated with HDT and dispersed in *n*-decane at 50 °C in the presence of R5S at 128 mg/mL after *t* = 0 min (a) and after 70 min at 50 °C (b). Solid lines: fits according to eq E12 in the Supporting Information. Dashed lines: deconvoluted contributions to the model, as indicated in the graphs.

After heating for 70 min (Figure 3b), the scattering pattern displayed a shallow slope at intermediate *q*-values (up to 0.1 Å^–1^), marking the presence of elongated structures. Again, a combination of a form factor of spherical particles and of dumbbell-shaped particles satisfactorily modeled the data. A Porod term was now required to model excess scattering at small *q*-values, indicating that larger aggregates were present at this stage. 49% of the particle core volume occupied by spherical primary and dumbbell-shaped particles combined were primary nanoparticles that did not undergo aggregation. However, as aggregates containing more than 2 nanoparticles are not accounted for in this estimation, the overall fraction of primary nanoparticles is lower at this stage, meaning that significantly more primary particles underwent aggregation than after 0 min.

The scattering patterns obtained after 150 min of heating (Figure 2b) were marked by strong forward scattering, indicating that aggregates containing many primary nanoparticles dominated the dispersion. A shoulder that emerged at approximately 0.1 Å^–1^ is likely due to a structure factor peak that indicates superstructures consisting of primary particles with regular spacings that may be partially fused, in agreement with results from TEM. The dispersion contained large particles with a wide range of geometries. Equation 1 became unsuitable and the data were not amenable to model-based fitting.

### Effect of the Temperature on AuNP Coarsening in the Presence of R5S

The time-dependent scattering from AuNP dispersions with R5S at 128 mM at 40 and 60 °C are shown in Figure S6 in the Supporting Information. The evolution of scattering at all temperatures was qualitatively similar, but changes occurred at different times. We determined time-dependent power law exponents *m* from the scattering patterns in Figure S6 at *q*-values between 0.013 Å^–1^ and 0.045 Å^–1^, corresponding to length scales of approximately 10 to 50 nm, using power-law fits according to function E7 in the Supporting Information. They are shown in log–log representation in Figure 4 for all investigated temperatures. At all temperatures, the exponent *m* was constant and slightly below 0 at early times, originating from the intensity-plateau of the form factor of primary particles and dumbbells at small *q*-values (Figure 3a). *m* started to decrease after 20 min at 60 and 50 °C and after 280 min at 40 °C. It reached −1.5 after 690, 150, and 40 min for 40 °C, 50 °C, and 60 °C, respectively. This corresponds to mass fractals with fractal dimensions of 1.5 over the probed length scales,^49^ indicating the formation of branched aggregates.

**Figure 4:**
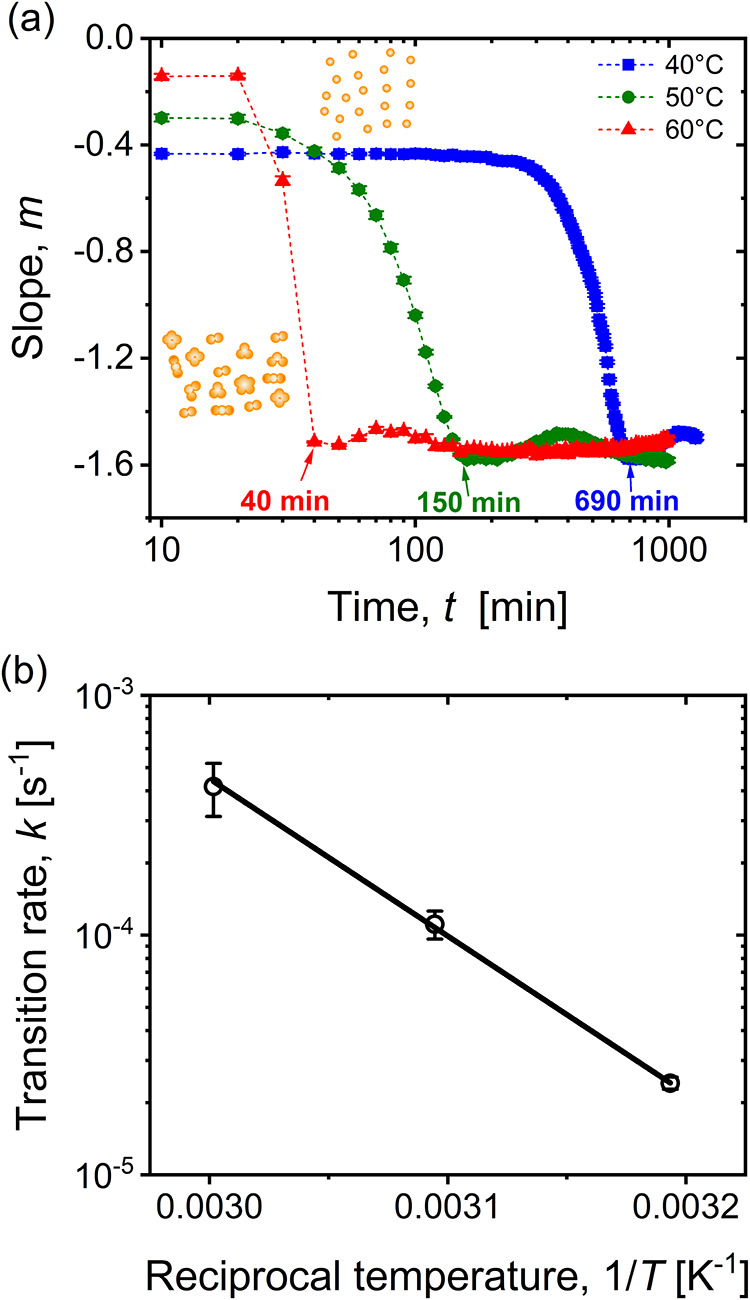
(a) Slope *m* of the time-dependent SAXS scattering in the *q*-range 0.013 Å and 0.045 Å for 40 (blue), 50 (green), and 60 °C (red symbols). (b) Times at which *m* reached −1.5. Solid line: Arrhenius fit (eq E17 in the Supporting Information).

The continuous decrease in slope reflects the progressive aggregation of primary particles into larger structures. Once the slope stabilized around −1.5, the structure at the probed length scales (approximately 10 to 50 nm) remained unchanged. At this point, the majority of primary particles were within aggregates that did not change their structure at length scales of approximately 10 to 50 nm. Further growth occurred predominantly through continuous aggregation of existing aggregates rather than primary particles. We define *t*_f_ as the time at which the final slope was reached and the majority of the primary particles had coarsened significantly by aggregation.

We used the time-dependent scattering results to assess the kinetics of nanoparticle aggregation and identify rate-limiting steps. First, let us assume that aggregation is limited by diffusion, such that the aggregation rate depends solely on the diffusivity of the primary particles. In this case, the time *t*_*x*_ required to decrease the number of primary particles to a given fraction, *x* = *N*(*t*_*x*_)/*N*_0_, can be approximated by

2 $$t_{x}\propto \frac{\eta }{k_{B}T}$$

with η the dynamic viscosity of the solvent, *k*_B_ the Boltzmann constant, *T* the temperature and *N*_0_ the initial number concentration of primary particles (see the Supporting Information for more details).

Increasing temperature increases the diffusion coefficient of primary particles directly and by decreasing solvent viscosity,^50,51^ effectively shortening the time between collisions. The expected rate change from 40 to 50 °C would imply a decrease of *t*_*x*_ by a factor of 1.13, while increasing the temperature from 40 to 60 °C would decrease it by 1.28. Experimentally, we found that *t*_f_ decreased much faster, by a factor of 4.6 from 40 to 50 °C and by a factor of 17.3 from 40 to 60 °C. We conclude that the aggregation of nanoparticles in the early stages is not limited by diffusion. A different energy barrier hindering fusion of particles limits the rate of the overall process.

An Arrhenius fit (see eq E17 in the Supporting Information) of *t*_f_ is shown in Figure 4b. The exponential change of *t*_f_ suggests that aggregation is controlled by a single process with an activation energy of 126 ± 4 kJ mol^–1^. This value is slightly below that of the binding energy of thiol on gold surfaces (130 to 210 kJ/mol^52,53^ ) and presumably in the same range as the HDT-R5S exchange energy on the AuNP surfaces. Therefore, it seems reasonable to assume that the exchange reaction of HDT to R5S on the gold surfaces determines the rate of nanoparticle aggregation: An increase in temperature speeds up the process because it reduces the adsorption affinity of both the sulfur and thiol species to the gold surface,^33^ enabling stronger exchange of HDT by R5S, which is present in excess. In the following, results from SANS, TGA, and SAXS are presented to investigate different additive concentrations to characterize how the ligand exchange rate affects the structural integrity of the shell.

### Influence of Additive Type on AuNP Coarsening

We tested the effect of other additives and added the cyclic sulfide thionane (R9S) and the cyclic amine pyrrolidine (R5N) to the same dispersions. The goal was to investigate the influence of molecular size and functional group, respectively, on temperature-induced coarsening of AuNPs. For this, temperature-dependent SAXS measurements were performed during heating from 36 to 100 °C, shown in Figure S8 in the Supporting Information.

Scattering from dispersions with a gold concentration of 10 mg/mL containing R5S at a concentration of 512 mM continuously changed during heating in a way that was similar to that at constant temperature (Figures 2 and S8 in the Supporting Information). Forward scattering increased at ∼50 °C, and a weak structure factor peak at 0.1 Å^–1^ appeared at ∼70 °C, both indicating that aggregation sets in. At temperatures above 80 °C, scattering at *q*-values above 0.03 Å^–1^ clearly decreased, pointing to sedimentation of aggregates. Furthermore, the form factor peak at 0.3 Å^–1^ and the structure factor peak at 0.1 Å^–1^ disappeared, indicating that the primary nanoparticles within aggregates fused together.

Adding R5N or R9S as additives at equal concentration (512 mM), the scattering curves did not indicate coarsening of the AuNPs at temperatures up to 100 °C. At low temperatures, weak deviations from a form factor of polydisperse spheres at small *q*-values point to agglomeration of a small fraction of AuNPs, which was not observed for additive-free dispersions (also shown in Figure S8 in the Supporting Information). With increasing temperature, however, the deviations diminished and only dispersed AuNPs were observed. It can be concluded that R5N and R9S are unable to induce aggregation of AuNPs at temperatures below 100 °C.

### Ligand Shell Composition and Structure

Earlier MD simulations have shown that R5S replaces HDT on the AuNP surfaces,^46^ possibly deteriorating the protective function of the ligand shell. R9S molecules, in contrast, are too large to reach the gold surface through a dense ligand shell. R5N is able to reach the surface, but its binding energy is insufficient for exchanging HDT on the gold surface. Consequently, ligand exchange of HDT by R9S and R5N is presumably less pronounced than ligand exchange by R5S, retaining the protective function of the shell, also at elevated temperatures.

To investigate this, SANS was carried out to quantify the structure and composition of the ligand shell in the presence of additive molecules. HDT-coated AuNPs were dispersed in deuterated *n*-decane (decane-*d*_22_) at a gold concentration of 10 mg/mL to obtain sufficient scattering intensities. R5S, R9S or R5N were added at room temperature at concentrations of 0, 64, 256, and 512 mM to expose the shells to various amounts of additive molecules. The samples were stored for 2 days prior to the measurements to allow for an absorption/desorption balance on the gold surface to set in. A measuring temperature of 35 °C was chosen to initiate additive-induced changes in the ligand without the occurrence of strong AuNP coarsening that disrupts the ligand shells entirely.

Figure 5a shows SANS patterns of AuNP dispersions at different R5S concentrations. All scattering patterns featured a shoulder above 0.02 Å^–1^ and a faint peak at 0.15 Å^–1^, in accordance with the scattering pattern expected for spherical core–shell particles. Additionally, weak forward scattering was present in all cases, indicating weak agglomeration of the AuNPs, in agreement with temperature-dependent SAXS measurements (Figure S8 in the Supporting Information). The overall appearance of the SANS patterns of AuNPs in the presence of R5N and R9N was similar (Figure S10 in the Supporting Information); most notable are additive-dependent differences in the absolute intensities of the shoulder.

**Figure 5:**
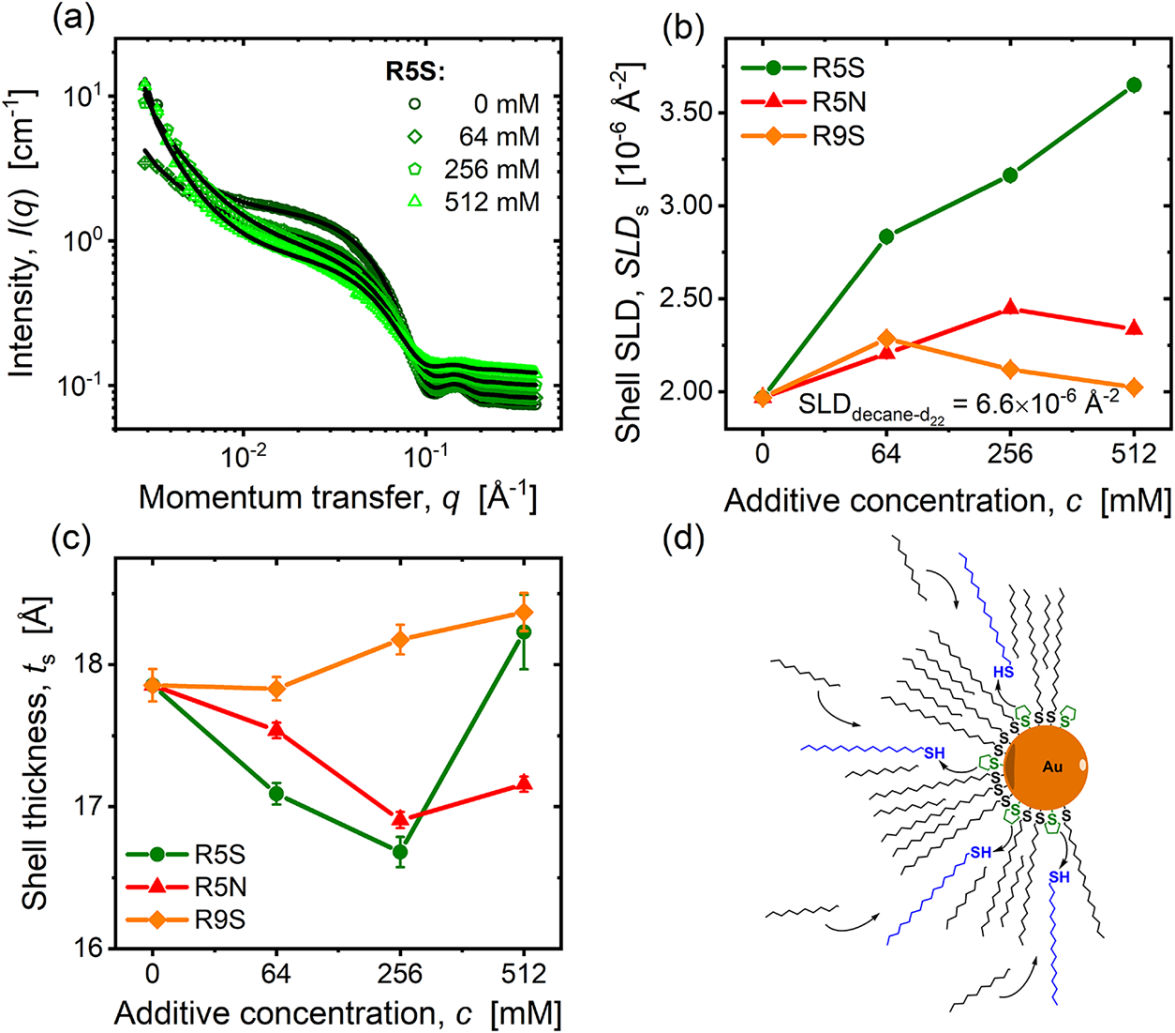
(a) SANS patterns of AuNP dispersions at a gold concentration of 10 mg/mL in the presence of R5S at concentrations as indicated in the graph. The black lines are model fits according to eq 3. (b) Scattering length density, *SLD*_s_, and (c) thickness of the shell, *t*_s_, in dependence on additive concentration for R5S (green data), R5N (red data) and R9S (orange data). (d) Sketch of the ligand exchange mechanism: HDT is replaced by R5S on the surface, opening up space for decane to enter the shell. As a result, *SLD*_s_ increases.

The absolute scattering intensities depend on the contrast between different components in the system: While the scattering length densities of the gold core and the solvent are set, that of the ligand shell depends on its composition and, therefore, on the specific interactions of the additive molecules with the ligand shell. To deduce the composition of the ligand shell, the SANS patterns were modeled using the equation

3 $$I_{\mathrm{tot}}\left(q\right)=I_{\mathrm{Porod}}\left(q\right)+I_{\mathrm{pCS}}\left(q\right)+I_{\mathrm{OZ}}\left(q\right)+I_{\mathrm{bkg}}$$

with *I*_pCS_(*q*) a form factor for polydisperse spherical core–shell particles to describe the core and shell of the AuNPs, which includes the shell thickness *t*_s_ and the scattering length density *SLD*_S_ of the shell as free variables. *I*_OZ_(*q*) is an Ornstein–Zernike structure factor to describe scattering from composition fluctuations in the solvent and *I*_Porod_(*q*) a Porod term to account for weak agglomeration of the AuNPs. Lastly, *I*_bkg_ accounts for incoherent background scattering. Full equations are given in eqs E27–E30 and illustrated in Figure S9, both in the Supporting Information. For R5N and R9S, excellent comparisons between the model and data were obtained. In the case of R5S, slight deviations were observed, most probably caused by the onset of AuNP aggregation.

*SLD*_s_ is a measure of the shell composition. It is particularly sensitive to the presence of deuterated *n*-decane within the shell, as its scattering length density (6.6 × 10^–6^ Å^–2^) differs significantly from that of the nondeuterated HDT ligands or additive molecules (all in the range of −0.3 × 10^–7^ Å^–2^ to −3 × 10^–7^ Å^–2^). In the absence of additive molecules, *SLD*_s_ was 1.97 × 10^–6^ Å^–2^ (Figure 5b). This corresponds to a shell composition of 56:44 n/n HDT to *n*-decane, assuming bulk molecular volumes for both species. Upon addition of additive molecules, *SLD*_s_ increased for all additive types. R5S led to the largest measured *SLD*_s_ of 6.65 × 10^–6^ Å^–2^ at 512 mM. In contrast, the presence of R5N and R9S increased *SLD*_s_ only weakly. At 256 mM R5N it was 2.45 × 10^–6^ Å^–2^ before decreasing again at higher concentrations. The larger additive R9S only led to an increase up to 2.29 × 10^–6^ Å^–2^ at 64 mM, followed by a weak decrease at higher concentrations.

Figure 5c shows *t*_s_ in dependence on additive concentration for each investigated additive type. In the absence of additive molecules, the shell thickness was 17.9 Å ± 0.2 Å, slightly below the length of fully stretched HDT ligands.^20^ This indicates that the ligands have an extended conformation and that they are homogeneously distributed in the shell. With increasing additive concentration, marginal differences in *t*_s_ below ∼1 Å were observed. This points to weak structural changes of HDT ligands within the shell during the presence of additive molecules of any type, while remaining in a stretched conformation.

The strong increase in *SLD*_s_ in combination with a marginally changing *t*_s_ upon the addition of R5S is consistent with a partial exchange of HDT by the smaller additive molecule on the gold surface. This exchange frees up space, allowing more *n*-decane molecules to solvate the ligand shell. Consequently, the fraction of *n*-decane in the shell increases, leading to a larger *SLD*_s_. For R5N and R9S, the minimal increase in *SLD*_s_ at low concentrations is likely due to a limited ligand exchange. At higher concentrations, the observed reduction of *SLD*_s_ points to physisorption of R5N or R9S that may crowd the shell, displacing *n*-decane and reducing *SLD*_s_. These results were confirmed by TGA. Assuming that all unbound molecules were removed during sample preparation and that each R5S molecules replaces between 1 and 2 HDT ligands within the shell (see the Supporting Information on a more detailed discussion on these assumptions), we found that 128 mM of R5S replaced 21% of the HDT (in a dispersion with a gold concentration of 2.5 mg/mL) after 1 day at room temperature and 82% after 5 h at 50 °C (see Figure S7a in the Supporting Information). In the case of R5N and R9S, no indication for ligand exchange was found (see Figure S7b in the Supporting Information).

It is possible for R5S to reach the gold surface, and it has affinity to it. The law of mass action causes replacement of HDT by the excess R5S. This likely weakens the protective function of the ligand shell, which allows gold surfaces of different particles to come closer to each other, initiating aggregation. To confirm this, temperature-resolved small-angle X-ray scattering results were performed as discussed in the following.

### Additive-Dependent Core Surface Spacing

Alkylthiols form dense ligand shells with thicknesses that depend on the ligand length.^48,54,55^ Partial exchange of HDT by R5S in the AuNP ligand shell may reduce the spacing between gold surfaces in agglomerates. We used controlled partial ligand exchange to replace HDT ligands by R5S without inducing nanoparticle coarsening. To this end, we treated AuNPs with R5S at concentrations of 64 mM or 128 mM for 24 h and purified them to obtain AuNPs where either 4% to 8%^46^ or 12% to 21% of HDT had been replaced by R5S (see Figure S7 in the Supporting Information). The dispersions were cooled using well-established protocols to induce AuNP agglomeration^47,48^ while X-ray scattering patterns were obtained at different temperatures. Here, SAXS was used to enable direct comparison with the coarsening experiments, as the spacing between gold surfaces in TEM measurements can be altered by drying effects and by the arrangement of AuNPs into monolayers rather than three-dimensional agglomerates.^21^ Toluene is used as solvent, because its freezing point is far below the agglomeration temperature of AuNPs. The core surface spacings *s* were calculated using eq E11 in the Supporting Information; they are shown in Figure 6 as a function of temperature in the agglomerated state.^21^

**Figure 6:**
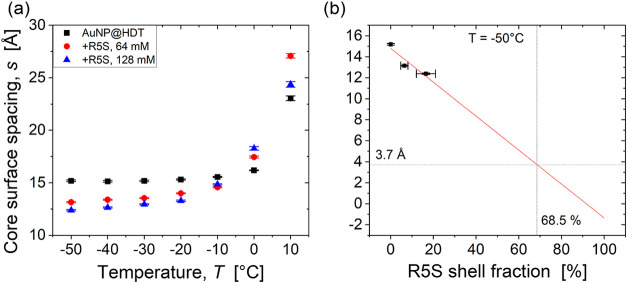
Temperature-dependent core surface spacings, *s*, from SAXS for additive-free reference AuNP@HDT (black symbols) and particles treated with R5S of 64 mM (red symbols) and 128 mM (blue symbols) in toluene (a). Linear approximation of the spacing as a function of the R5S fraction in the shell using the spacings of (a) at −50 °C (b).

*s* decreased with temperature for all shell compositions, probably due to the increasing bundling of the ligands that allows particles to pack more densely.^47,48^ The AuNP@HDT reference reached a spacing of 15.2 Å at −50 °C. Modification with 64 mM R5S (6.4% of R5S in the shell) led to a spacing of 13.2 Å at −50 °C, while 128 mM (16.5% of R5S in the shell) led to a spacing of 12.4 Å at −50 °C.

We propose that the replacement of the larger HDT in the particles’ shells by smaller R5S causes the reduced spacing, which in turn enables joining of the metal surfaces. This is consistent with literature: For example, Bae and colleagues investigated the approach of gold nanoparticle cores with pure hexadecyltrimethylammonium bromide (CTAB) shells and mixed shells of CTAB and the smaller octylamine during their aggregation. The core-surface spacing for particles coated with pure CTAB decreased continuously from 4 to 1 nm before joining, while cores with mixed shells approached abruptly. For both systems, at a critical distance of approximately 1 nm, a jump-to-contact occurs, after which both cores join. It was concluded that the enhanced mobility of mixed shells promotes the rearrangement and detachment of ligands in the gap region, leading to the more rapid aggregation of the nanoparticles.^56^

Note that we could not measure the core surface spacings *s* of our particles for high R5S fractions. Their distribution became too wide for analysis via SAXS when aggregation set in. Figure 6b shows the three measured spacings at R5S shell fractions below 25%. We can linearly extrapolate and estimate that spacings would drop to below 1 nm at R5S shell fractions above 40%. We can use the same approach to roughly estimate the spacing at the R5S fraction of 68.5% that was reached after 5 h at 50 °C (Figure S7a in the Supporting Information); this would be approximately 4 Å.

Literature suggests that cores of gold nanoparticles with surface spacings in the range of 0.5 to 2 nm can form metal connections, depending on the system.^56–59^ Two mechanisms are discussed. In one, ligand molecules are displaced from the contact by desorption of rearrangement, allowing direct contact and adhesion of the cores.^57–60^ A second mechanism involves the diffusion of gold atoms that form a bridge between cores^61–63^ even if the ligands are not (entirely) displaced. We propose that, in our case, R5S reduces the spacing between gold surfaces and is more easily displaced than HDT, as the sulfide is less strongly bound to the gold. Thus, R5S initiates its own removal by reducing *s* to below its critical value.

## Conclusion

Coarsening of apolar gold nanoparticles is hindered by the steric repulsion of ligand shells that prevents the cores from coming close. Consequently, thermally induced coarsening typically takes place at temperatures above 120 °C, where the ligand desorption leads to a structural defect in the ligand shell. Here, we showed that chemical additives can be used to provoke structural defects by ligand exchange reactions, leading to coarsening at near-ambient conditions.

Transmission electron microscopy and small-angle X-ray scattering revealed that adding the cyclic sulfide additive molecule tetrahydrothiophene (R5S) to a dispersion of hexadecanethiol (HDT)-coated gold nanoparticles (AuNPs) induces aggregation and joining of cores at temperatures of 40 to 60 °C. Initially, AuNPs form small aggregates consisting of only a few AuNPs. These grow over time and form micron-scale aggregates after several hours. Concurrently, metal cores form metallic connections.

An Arrhenius analysis of AuNP aggregation identified the exchange of HDT on the gold surfaces by R5S as the rate-limiting step of the overall process. Small-angle neutron scattering indicated that increasing R5S concentration leads to larger fractions of R5S in the forming mixed monolayer on the nanoparticle surface; thermogravimetric analysis confirmed that the fraction at a given time is temperature-dependent. Particle cores join when a certain fraction of R5S in the shell is reached. In contrast, cyclic sulfide thionane (R9S) or cyclic main pyrrolidine (R5N) did not induce aggregation or joining of AuNP. None of them was able to replace sufficiently large fractions of HDT in the ligand shell.

We identified the core surface spacing as the main factor in the joining of metal particle cores. Temperature-induced agglomeration of AuNP with mixed HDT-R5S monolayers at defined ratios in SAXS indicated that larger R5S fractions reduce the spacing between the gold cores in aggregates. There exist a certain critical spacing (likely below 1 nm) below which the metal cores form metallic joints.

Our work provides a pathway for controlled coarsening of apolar gold nanoparticles under mild conditions. As opposed to their reversible agglomeration, this allows for large changes in interparticle coupling, e.g., electron transfer or plasmonic interactions, that lead to dramatic changes in the system’s properties. It paves the way for improved or new materials in fields such as printed electronics and sensing.

## Materials and Methods

### Nanoparticle Synthesis

Gold nanoparticles (AuNPs) with a core diameter of 4 nm were synthesized following a method described previously.^47^ In short, a mixture of 9 mL of *n*-pentane (Carl Roth, 99%) and 9 mL oleylamine (Sigma Alrich, technical grade, 80–90%) was added to 100 mg of tetrachloroauric­(III) acid trihydrate HAuCl_4_·3H_2_O. The mixture was stirred (with a magnetic stirrer bar) for 21 min at 22 °C under an argon atmosphere. Then, a mixture of 1 mL *n*-pentane, 1 mL of oleylamine and 40 mg *tert*-butylamine borane complex (Fluka, 97%) was added to the gold precursor solution. The mixture was stirred for 60 min. The dispersion was purified by precipitation with 50 mL of a mixture of ethanol and methanol (ratio 3:2) and centrifugation at 2000 rpm (689 rcf) for 1 min. The supernatant was removed, and the nanoparticles were redispersed in 2.5 mL toluene (gold concentration of 10 mg/mL).

### Ligand Exchange

For the ligand exchange the dispersion was heated to 80 °C, followed by the addition of 1 mL of hexadecanethiol (Sigma-Aldrich, GC, 95%) (0.4 mL per 1 mL of a dispersion with a gold concentration of 10 mg/mL). The mixture was stirred for 15 min at 300 rpm. The dispersion was purified by precipitation with 3 times the sample volume (3 mL for 1 mL dispersion) of a mixture of ethanol and methanol (ratio 2:1) and centrifugation at 2000 rpm (689 rcf) for 5 min. The supernatant was removed, and the nanoparticles were redispersed in 2.5 mL toluene. The washing step was repeated once with a cooled mixture of ethanol and methanol (2:1). After the last washing step, the sample was redispersed in *n*-decane and diluted to a gold concentration of 2.5 mg/mL.

### Synthesis of Thionane (R9S)

The synthesis of the additive thionane was based on the protocol described by Singh et al.^64^ and described in detail in our earlier work.^46^

### Addition of Additives

Tetrahydrothiophene (R5S, Sigma-Aldrich, GC, 99%), thionane (R9S, own synthesis) and pyrrolidine (R5N, Sigma-Aldrich, GC, 99%) were used as additive. Before their addition to the AuNP dispersions, the AuNP dispersions at a gold concentration of 2.5 mg/mL in *n*-decane were heated to 50 °C for 10–15 min to ensure that all AuNPs were dispersed. Subsequently, the dispersions were cooled to 30 °C and the additives were added. The mixtures were shaken for 1–2 min minutes and stored without stirring for 24 h at room temperature.

### Small-Angle X-ray Scattering

A laboratory-scale Xeuss 2.0 instrument (Xenocs SA, France) was used for all SAXS measurements. The X-ray beam was produced by a copper K_α_ source (wavelength 1.54 Å) and focused on the sample with a spot size of 0.25 mm^2^. The measurements were performed with a sample–detector distance (SDD) of 1020 mm. The measurable momentum transfer *q* ranged from 0.01 Å^–1^ to 0.6 Å^–1^, with *q* being defined as *q* = 4π sin­(θ/2)/λ, were θ is the scattering angle. The samples were measured in borosilicate capillaries with a diameter of 1.5 mm.

Time-dependent aggregation measurements using AuNP dispersions with a gold concentration of 2.5 mg/mL and an additive concentration of 128 mM were performed at 40, 50, and 60 °C. Prior to each, a SAXS pattern was acquired at room temperature. After heating to the desired temperature, subsequent measurements with an acquisition time of 5 min per performed with a waiting time of 5 min after each measurement. Measurements were performed until no further changes were visible in the scatter curves.

Temperature-dependent aggregation measurements were performed using AuNP dispersions with a gold concentration of 10 mg/mL and an additive concentration of 512 mM. For these measurements, R5S, R5N, and R9S were used as additives. As a reference, an additive-free dispersion was measured. All samples were heated from 36 to 100 °C in steps of 2 K, with a waiting time of 5 min after each step that was followed by the acquisition of a SAXS pattern with an integration time of 5 min.

Temperature-dependent agglomeration measurements were performed similarly to our previous work.^21^ AuNP dispersions with a gold concentration of 2.5 mg/mL and R5S concentrations of 0 mM, 64 mM, and 128 mM were cooled from 20 °C to −50 °C in steps of 10 K. At each temperature, three SAXS patterns were measured with acquisition times of 5 min, following a 5 min waiting time.

The analysis of the time- and temperature-dependent scattering curves is described in the Supporting Information.

### Small-Angle Neutron Scattering

SANS measurements were performed at the Institute Laue-Langevin in Grenoble (France) at the instrument D22. Using a neutron wavelength of λ = 6 Å with a spread of Δλ/λ = 0.10 and sample–detector distances (SDD) of 17.6 m (detector 1) and 1.4 m (detector 2), an accessible *q*-range from 0.003–0.4 Å^–1^ was covered. The samples were measured in cylindrical quartz glass cells (type QS-120, Hellma, Germany) with a layer thickness of 2 mm. The obtained data were corrected for scattering by the empty cell and for parasitic scattering using a measurement of boron carbide. H_2_O was measured to calibrate the detector sensitivity. The measured data were converted to an absolute scale using a direct beam measurement, and the scattered intensity *I*(*q*) was obtained by azimuthally averaging the 2D scattering patterns.

AuNP dispersions with a gold concentration of 10 mg/mL dispersed in decane-*d*_22_ were measured in the presence of R5S, R5N, and R9N at concentrations of 128 mM, 256 mM, and 512 mM. Prior to the measurements, the samples were heated for 10 min at 36 °C, followed by a SANS data acquisition for 30 min. Scattering from the additive solutions (in the absence of AuNPs) was measured as a reference.

### Transmission Electron Microscopy

For the imaging of the gold nanoparticles a JEOL JEM2100 LaB6 electron microscope (JEOL Ltd. Tokyo, Japan) with 200 kV acceleration voltage, 0.14 nm line resolution, and a Gatan Orius SC1000 camera (Gatan Inc. Pleasanton, CA, USA) in brightfield mode was used. AuNP samples were prepared by drop-casting of 10–20 μL of the particle dispersion in *n*-decane (2.5 mg/mL) on a TEM copper grid on top of a clean room sheet for a fast removal of excess liquid. The sample was dried for 30 min.

For the characterization of the AuNP coarsening kinetics, a dispersion with a R5S concentration of 128 mM was heated to 50 °C. After 70, 150, and 300 min, samples were taken. These were drop-casted on a copper grid and analyzed using TEM. The analysis of the TEM images is described in the TEM section in the Supporting Information.

### Thermogravimetric Analysis

For the thermogravimetric analysis (TGA), additive-free and additive-containing nanoparticle dispersions with gold concentrations of 20–30 mg/mL were prepared. The additive-free dispersion was transferred to *n*-hexane after the second washing step following the ligand exchange. The additive-containing dispersions were washed twice after the addition of the additive to remove all unbound HDT and R5S molecules and redispersed in *n*-hexane at a gold concentration of 20–30 mg/mL. Due to the washing, the additive concentration is unknown.

The samples for TGA measurements were prepared by drop-casting the dispersions onto Al_2_O_3_ crucibles. *n*-hexane was evaporated at ambient conditions. Subsequently, the samples were dried in a vacuum oven (*p* = 10 mbar, *T* = 30 celsius) for 1–2 h. For the TGA measurement, an STA 449F3 setup from NETZSCH was used. The samples were heated with 10 °C/min to 1000 °C. During the heating, an argon atmosphere was used.

## Supplementary Material



## Notes

Characterization of particle size; characterization of the agglomeration fraction; characterization of the aggregation process using SAXS; thermogravimetric analysis of additive-free and additive-containing nanoparticle systems; characterization of the shell structure using SANS (PDF)

## ∥

Faculté de Médecine de Grenoble, Université Grenoble Alpes, Domaine de la Merci, 38700 La Tronche, France and Centre Hospitalier Universitaire Grenoble Alpes, CS10217, 38043 Grenoble Cedex 9, France
