# Supplementary material for: Reactive Additive-Induced Joining of Apolar Thiol-Coated Gold Nanoparticle Cores at Low Temperatures
Source: Langmuir. 2026 Jun 9;42(24):17260–70. doi: 10.1021/acs.langmuir.6c00514 (PMC13296492; doi:10.1021/acs.langmuir.6c00514)
Supplement: Supplementary file 1 [file la6c00514_si_001.pdf]

Supporting Information for: Reactive Additive-Induced Joining of Apolar Thiol-Coated Gold Nanoparticle Cores at Low Temperatures

Tobias Knapp,[†] Bart-Jan Niebuur,[†] Olga Matsarskaia,[‡] and Tobias Kraus[*,†,¶]

†*INM – Leibniz Institute for New Materials, Campus D2 2, 66123 Saarbrücken, Germany*

‡*ILL - Institut Laue Langevin, 71 Avenue des Martyrs, 38042 Grenoble, France*

¶*Colloid and Interface Chemistry, Saarland University, Campus D2 2, 66123 Saarbrücken, Germany*

E-mail: tobias.kraus@leibniz-inm.de

Number of pages: 20

Number of figures: 10

Number of tables: 4

Number of equations: 35

Transmission electron analysis

Transmission electron microscopy (TEM) measurements were performed to determine the size and shape of AuNPs. Figure S1a shows a TEM micrograph of 4 nm AuNPs, directly after their ligand exchange with hexadecanethiol (HDT).

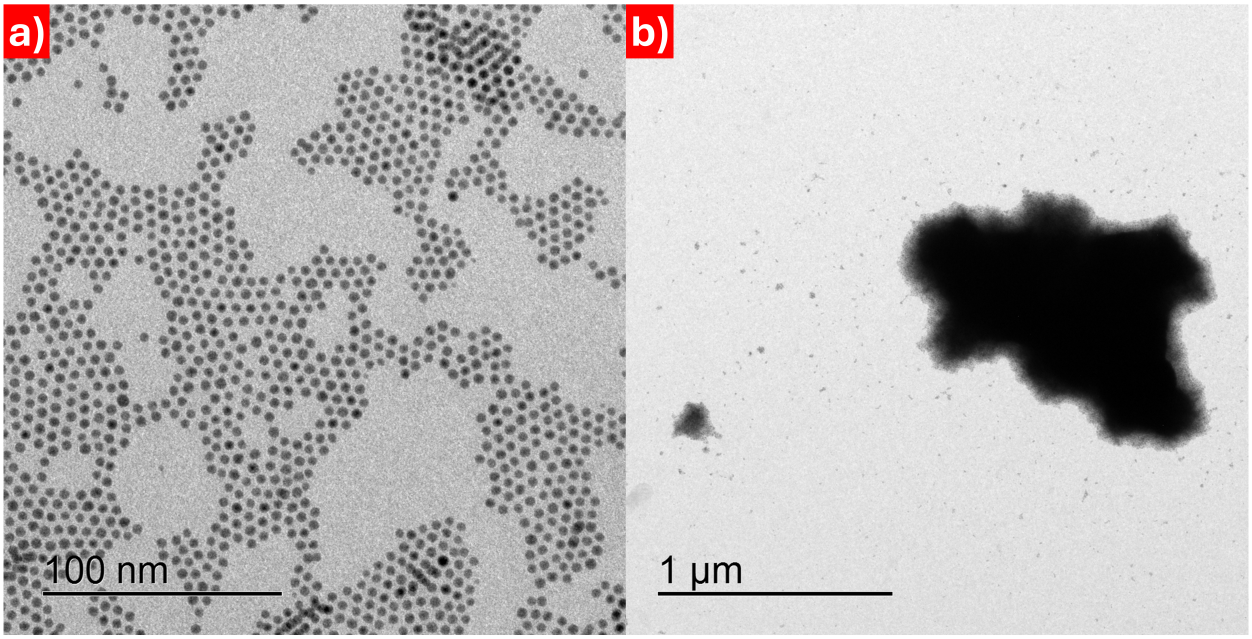

Figure S1: Transmission electron microscopy image of (a) 4 nm AuNP after the ligand exchange with HDT and (b) after the addition of R5S (128 mM) and heating at 50 °C for more than 300 min.

The particle diameter in the TEM images was determined in a stepwise image analysis using the software "Avizo" (Avizo 3D 2022.1, ThermoFisher Scientific, Waltham, Massachusetts, USA), similar to our previous work.[1] First, various filters ("Background Detection Correction", "greyscale filter", etc.) were applied to increase the contrast of the particles. Incomplete particles at the edge of the images were masked. The remaining particles were labeled using the function "Labeling". Using this method, the center-of-mass and the size of the particles were determined. The particle circularity was analyzed using the function:[2]

$$f_{\mathrm{circ}} = \frac{4\pi A}{P^2} \tag{E1}$$

with A the area (pixel count), and P the perimeter. The circularity describes how closely the 2D particle image approximates a circle. The parameter ranges from 0 to 1, with 1 representing the case of a perfectly spherical object.[2] The circularity of the particles before heating, shown in Figure 1a and d in the main text, was 0.86. We defined particles with a circularity of more than 0.6 as spheres.

Figure S2a shows a TEM micrograph of AuNPs modified with 128 mM of R5S and heated for 70 min at 50 °C. Its size distribution (Figure S2b) is broader than that of the primary particles before modification (Figure 1 in the main text), pointing to coarsening of the AuNPs by Ostwald ripening. As changes in the AuNP size dispersity could not be observed using SAXS, we assume that the coarsening by Ostwald ripening is mainly due to electron irradiation of particles during TEM measurements.

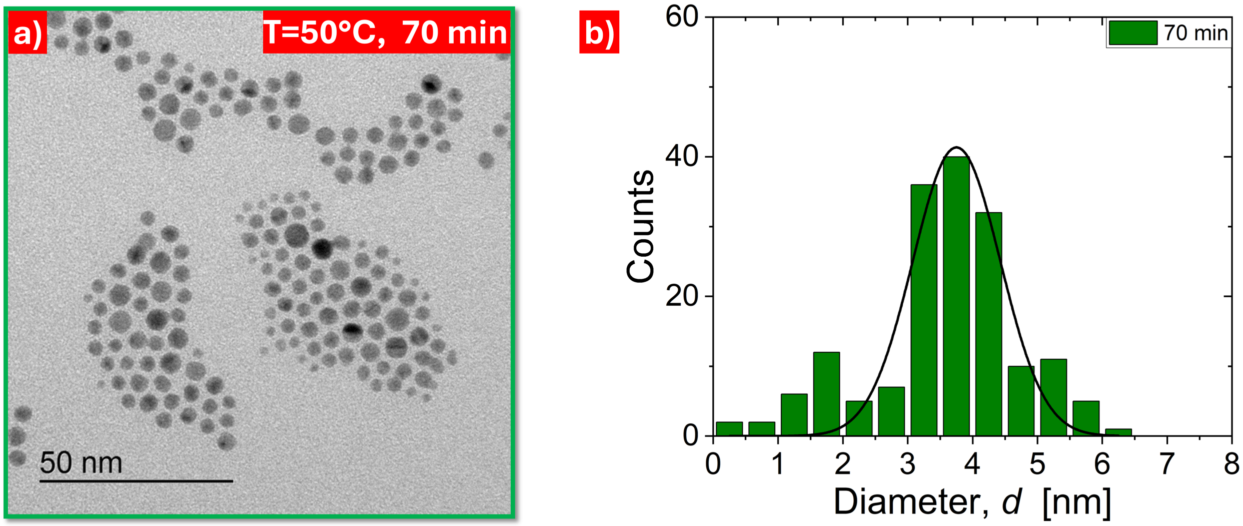

Figure S2: Coarsening of 3.8 nm gold nanoparticles coated with HDT dispersed in n-decane at 50 °C in the presence of R5S in a concentration of 128 mM. (a) Transmission electron micrograph shows the dispersion after 70 min at 50 °C. (b) Results of the size analysis of the micrograph.

The kinetic energy of electrons in our TEM experiments (200 keV) is much higher than the binding energy of gold atoms within AuNPs, which is typically in the order of a few eV.[3] Therefore, single atoms or clusters of atoms may be released from the particles to join other ones, resulting in AuNP coarsening by Ostwald ripening. Modification of the ligand shell by

R5S is expected to enhance this effect, because it weakens the protective effect of the shell.

In the work of Gutiérrez-Wing et al. and Azcárate et al., the effect of electron irradiation on the core geometry was analyzed.[4,5] For both thiol-coated and blank gold nanoparticles on a carbon substrate under electron irradiation, it was found that Ostwald ripening and coalescence occur within 5 min after the start of the electron irradiation.

Gutiérrez-Wing and co-workers found that ripening is energetically favored. They discovered that ripening involves the migration of ad-atoms and/or thiol-ad-atom clusters from smaller particles to larger ones. This process is based on the lower stability of ad-atoms on small cores due to the lower coordination resulting from the larger curvature. Additionally, they could demonstrate that the alkyl chains of the ligand can interact with the substrate through van der Waals forces, leading to the fixation of the particles. This fixation hinders the coalescence of the cores.[4]

Azcárate et al. could show that for coalescence, two equal planes (often the 100 planes) need to face each other, and the ligand coverage must be small. They could demonstrate that particles can reorient to align with specific planes. As a result, coalescence is favored for blank cores.[5] Bo et al. studied the heat-induced coalescence of oleylamine-coated gold particles using liquid-phase electron microscopy. They could demonstrate that coalescence was primarily induced by the heat treatment, rather than by the electron irradiation.[6] Therefore, we conclude that the irradiation of the electron beam is the main cause of the observed Ostwald ripening in this work.

Small-angle X-ray scattering (SAXS)

SAXS model fitting

The gold nanoparticles (AuNPs) used in this study were characterized using SAXS. Figure S4a shows the scattered intensity, $I(q)$, of AuNPs in dependence on momentum transfer, q, in their dispersed state, i.e., the scattering pattern represents the form factor of AuNPs.

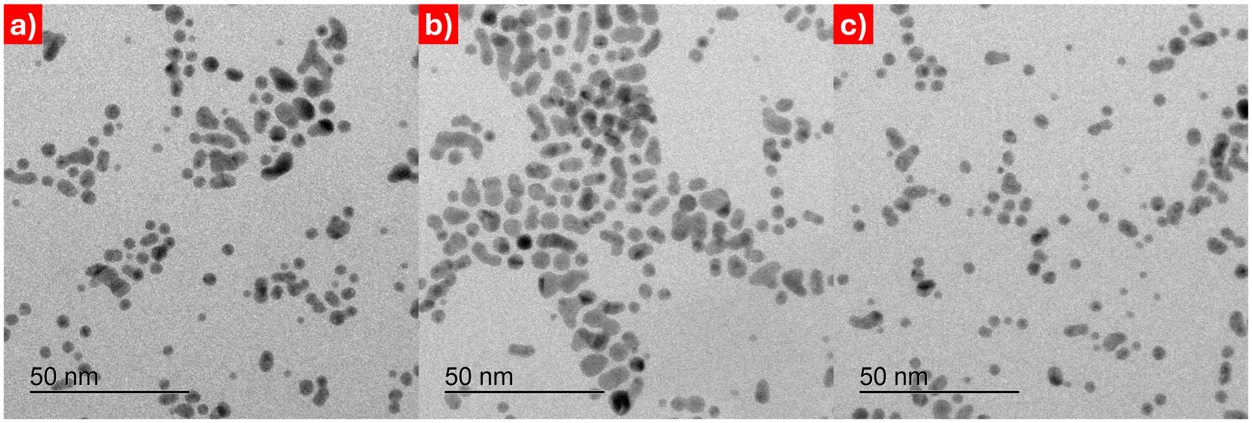

Figure S3: TEM micrographs (a-c) of HDT-coated 4 nm AuNP dispersed in n-decane with an additive concentration of 128 mM after heating at 50 °C for 150 min. These micrographs were used for the analysis of the sphericity and particle size shown in Figure 1e in the main manuscript.

As the scattering contrast between the organic ligands and solvent molecules is negligible, only the Au cores are visible. The size of the cores was determined by modeling the obtained scattering pattern using the expression

$$I(q) = I_{\mathrm{pS}}(q) + I_{\mathrm{bkg}} \tag{E2}$$

with $I_{\mathrm{pS}}(q)$ a form factor of polydisperse spheres[7] and I_{bkg} a constant accounting for background scattering. I_{pS} is given by

$$I_{\mathrm{pS}}(q) = C \int_0^{\infty} F_{\mathrm{S}}^2(q,r) r^6 G(r)\,\mathrm{d}r \tag{E3}$$

with $F_{\mathrm{S}}(q,r)$ the scattering amplitude of a sphere with radius r, $G(r)$ the distribution of radii, and C a scaling constant. $F(q,r)$ is given by

$$F_{\mathrm{S}}(q,r) = 3 \cdot \frac{\sin qr - qr\cos qr}{(qr)^3} \tag{E4}$$

$G(r)$ is approximated by a Gaussian distribution, given by

$$G(r) = \frac{1}{\sqrt{2\pi\sigma^2}} \exp\left(- \frac{(r - r_{\text{avg}})^2}{2\sigma^2} \right) \tag{E5}$$

with r_{avg} the average core radius and σ its standard deviation.

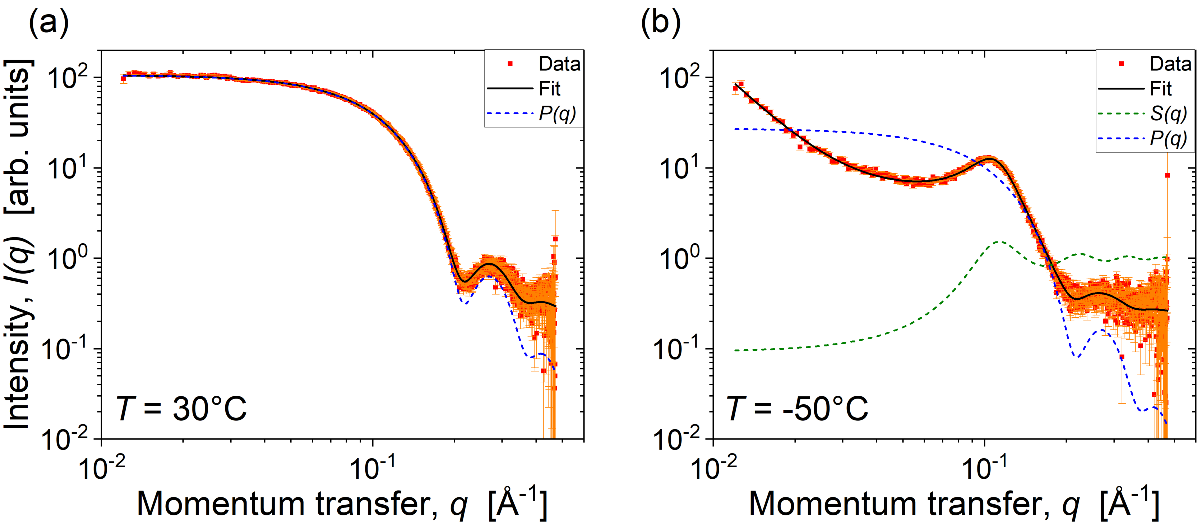

Figure S4: (a) SAXS pattern of 4 nm AuNPs dispersed in n-decane after the ligand exchange with HDT at 30 °C. Red dots: data. Black line: fit according to eq. E2. Blue dotted line: sphere form factor $P(q)$. (b) SAXS pattern of agglomerated AuNPs at -50 °C. Black line: fit according to eq. E6. Blue dotted line: sphere form factor $P(q)$. Green line: structure factor $S(q)$ for amorphous assemblies.

The AuNPs from all batches used in this study had an r_{avg} in the range of 3.8 − 4.1 nm and σ in the range of 10 − 11 %. The spherical shape of the AuNPs was confirmed using transmission electron microscopy, shown in Figure S1 for AuNPs after ligand exchange with HDT.

The temperature-dependent SAXS patterns (see Figure S5 and S4b) of the cooling experiments were analyzed following a method similar to that described by Hasan et al.[8] In this method, the SAXS patterns are modeled using the expression

$$I(q) = I_{\text{Porod}}(q) + I_{\text{pS}}(q) \left[1 - \chi_{\text{agglo}} + \chi_{\text{agglo}} \cdot S_{\text{HS}}(q)\right] + I_{\text{bkg}} \tag{E6}$$

Here, χ_{agglo} is the number fraction of AuNPs located inside agglomerates. $I_{\text{Porod}}(q)$ is a generalized Porod law,[9] accounting for scattering by the agglomerates, given by

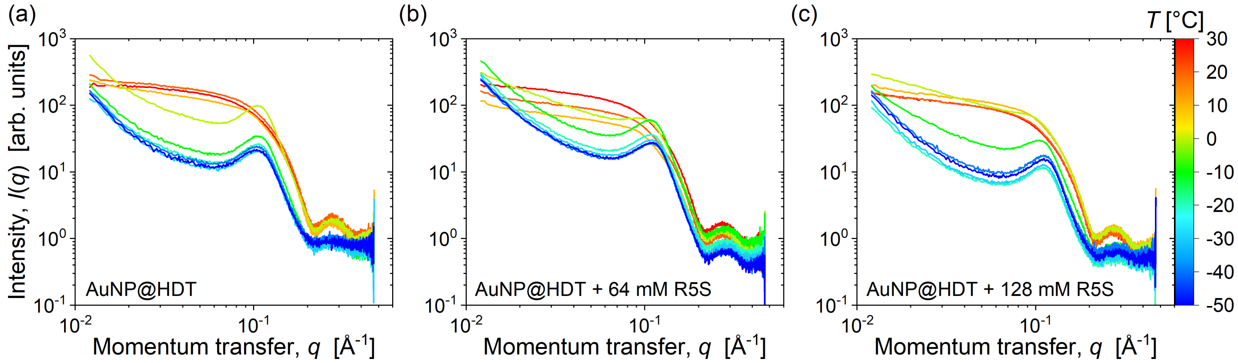

Figure S5: Temperature-dependent scattering curves of 4 nm coated with HDT (a) and modified with 64 mM (b) and 128 mM (c) of R5S dispersed in toluene during the cooling induced self-assembly.

$$I_{\text{Porod}} = \frac{B}{q^m} \tag{E7}$$

with B a pre-factor and m the Porod exponent, indicative of the fractal properties of the agglomerates.[10] $S_{\text{HS}}(q)$ is the Percus-Yevick hard-sphere structure factor[11] accounting for spatial correlation between AuNPs inside agglomerates. $S_{\text{HS}}(q)$ can be calculated as[7]

$$S_{\text{HS}}(q) = \frac{1}{1 + 24\eta_{\text{HS}} G_{\text{HS}}(2R_{\text{HS}}q)/(2R_{\text{HS}}q)} \tag{E8}$$

with R_{HS} the hard sphere radius and η_{HS} the hard-sphere volume fraction inside agglomerates. The function $G_{\text{HS}}(x)$ is defined as:

$$\begin{aligned} G_{\text{HS}}(x) = \gamma' \frac{\sin x - x\cos x}{x^2} + \delta' \frac{2x\sin x + (2 - x^2)\cos x - 2}{x^3} \\ + \epsilon' \frac{-x^4 \cos x + 4[(3x^2 - 6)\cos x + (x^3 - 6x)\sin x + 6]}{x^5} \end{aligned} \tag{E9}$$

Here, γ', δ' and ϵ' are help functions, given by

$$\gamma' = \frac{(1+2\eta)^2}{(1-\eta)^4}, \qquad \delta' = \frac{-6\eta(1+\eta/2)^2}{(1-\eta)^4}, \qquad \epsilon' = \frac{\eta\gamma'}{2} \tag{E10}$$

During fitting, r_{avg} and σ were kept fixed using the values obtained at the highest mea-

sured temperature of each respective run, i.e., where no agglomerates are present.

R_{HS} can be used to calculated the core surface spacing s between gold surfaces of neighboring particles in agglomerates as

$$s = 2(R_{\mathrm{HS}} - r_{\mathrm{avg}}) \tag{E11}$$

The model employed to analyze SAXS patterns during the coarsening process is based on a combination of a polydisperse sphere form factor, a polydisperse dumbbell form factor, and a Porod term, given by

$$I_{\mathrm{tot}}(q) = I_{\mathrm{Porod}}(q) + I_{\mathrm{pS}}(q) + I_{\mathrm{pDum}}(q) + I_{\mathrm{bkg}} \tag{E12}$$

The polydisperse dumbbell form factor, $I_{\mathrm{pDum}}(q,r)$, describes fused bodies consisting of two equally sized spheres with a Gaussian size distribution $G(r)$, and is given by

$$I_{\mathrm{pDum}}(q) = \frac{C}{V}\int_0^\infty P_{\mathrm{Dum}}(q,r) r^6 G(r) dr \tag{E13}$$

with C a scaling amplitude, V the total volume of the particle. $P_{\mathrm{Dum}}(q,r)$ the form factor of monodisperse dumbbells, given by

$$P_{\mathrm{Dum}}(q) = \int_0^{\pi/2} F^2_{\mathrm{Dum}}(q,r,\theta)\sin(\theta) d\theta \tag{E14}$$

which is the average of the scattering amplitude $F_{\mathrm{D}}(q,r,\theta)$ squared, over all orientations. $F_{\mathrm{D}}(q,r,\theta)$ is written as[12]

$$\begin{aligned} F_{\mathrm{D}}(q,r,\theta) = 4\pi r^3 \int_{-h/R}^{1} &\cos\left[q\cos\theta(rt+h)\right] \\ &\times(1-t^2)\frac{J_1[qR\cdot\sin\theta(1-t^2)^{1/2}]}{qR\cdot\sin\theta(1-t^2)^{1/2}}\mathrm{d}t \end{aligned} \tag{E15}$$

Here, $J_1(x)$ is the first-order Bessel function and h the distance between the center of the core and the cross-plane between the two spheres, r_n, given by

$$h = \sqrt{r^2 - r_\mathrm{n}^2} \tag{E16}$$

During the fitting, the sphere radius r in $P_\mathrm{pS}(q)$ and in $P_\mathrm{pDum}(q)$ were kept identical.

AuNP coarsening at 40 °C and 60 °C

The SAXS patterns obtained during AuNP coarsening at different times at 40 °C and 60 °C are shown in Figure S6.

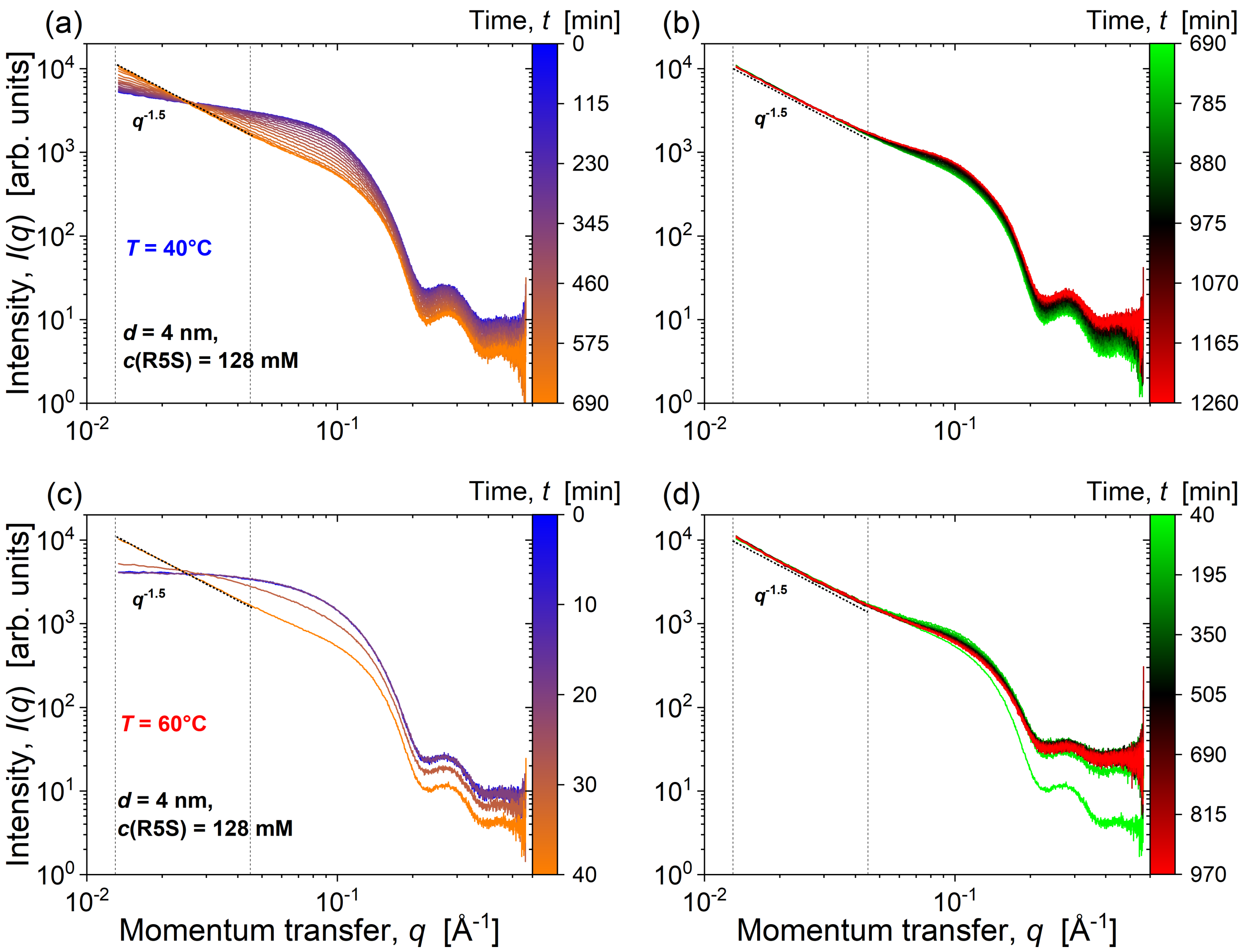

Figure S6: SAXS patterns of AuNPs, dispersed in n-decane with a R5S concentration of 128 mM during heating at 40 °C (a,b) and at 60 °C (c,d).

Arrhenius analysis of AuNP coarsening

The activation energy, E_{A}, of the coalescence process was calculated using the Arrhenius equation, given by

$$k = A \cdot e^{-\frac{E_{\mathrm{A}}}{RT}} \tag{E17}$$

with k the transition rate, determined as $1/t_{\mathrm{f}}$, R the gas constant and T the absolute temperature.

Thermogravimetric analysis (TGA)

TGA of R5S containing dispersions

Similar to our previous work, TGA was used to determine the composition of the ligand shells and the exchange behavior of the additive R5S.[13] Figure S7a shows the mass loss curves of the additive-free AuNP@HDT system and of the R5S containing particle system at a concentration of 128 mM, both stored for one day at room temperature and for 5 h at 50 °C. The overall shell mass decreased in the presence of R5S, which is attributed to the ligand exchange of the large HDT by the small R5S. In the case of R5N and R9S (Figure S7b), the shell mass increases after the modification with the additive, excluding a ligand exchange.

To quantify ligand exchange, the ligand shell density of the additive-free particle system must be calculated. The surface area A_{core} of one particle cores is

$$A_{\mathrm{core}} = 4\pi r_{\mathrm{core}}^2 \tag{E18}$$

The number of particles N_{AuNP} follows from the total gold mass m_{gold} remaining in the crucible after the TGA measurement as

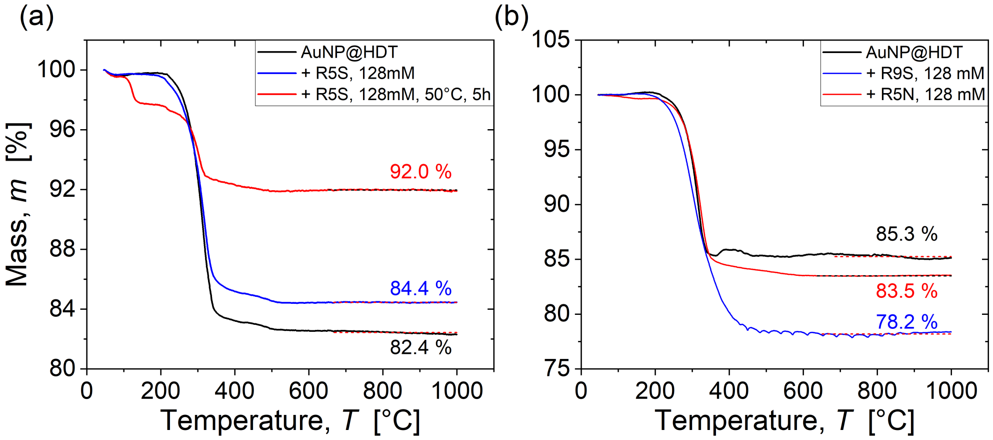

Figure S7: Results of the thermogravimetric analysis of different nanoparticle systems. (a) Mass loss of pure AuNP@HDT (black) and two AuNP@HDT samples with a R5S additive concentration of 128 mM after one day (blue) and after 5 h at 50 °C (red). (b) mass loss of pure AuNP@HDT (black) and AuNP@HDT modified with R9S at a concentration of 128 mM after one day of storage (blue), and with R5N at a concentration of 128 mM after one day of storage (red). The dotted lines indicate the areas of the curve used to determine the change in mass.

$$N_{\mathrm{AuNP}} = \frac{m_{\mathrm{gold}}}{m_{\mathrm{core}}} = \frac{m_{\mathrm{gold}}}{V_{\mathrm{core}} \cdot \rho_{\mathrm{gold}}} = \frac{m_{\mathrm{gold}}}{\frac{4}{3}\pi r_{\mathrm{core}}^3 \cdot \rho_{\mathrm{gold}}} \tag{E19}$$

With m_{core} the mass of a single AuNP core, V_{core} the volume of a single AuNP core and ρ_{gold} the density of gold. N_{AuNP} relates to the total core surface of all AuNPs in the crucible as

$$A_{\mathrm{total}} = A_{\mathrm{core}} \cdot N_{\mathrm{AuNP}}. \tag{E20}$$

The total number of surface-bound HDT ligands, N_{total}, is determined from the mass loss, m_{loss}, during the TGA measurement as

$$N_{\mathrm{total}} = \frac{m_{\mathrm{loss}}}{M_{\mathrm{HDT}}} \cdot N_{\mathrm{A}}, \tag{E21}$$

where M_{HDT} is the HDT molar mass (258.51 g/mol) and N_{A} is the Avogadro constant. Subsequently, the ligand shell density of the additive-free particle system, ρ_{LS}, is calculated

as

$$\rho_{\mathrm{LS}} = \frac{N_{\mathrm{ligand}}}{A_{\mathrm{total}}} \tag{E22}$$

It equals 6.71 nm^{-2}, in good agreement with previously published work on similar systems.[1,13]

In the following, the fraction of HDT that is exchanged during surface modification by R5S, a, is determined. From only the mass of the ligand shell, it is impossible to determine both the ligand density and ratio between ligands in mixed ligand shells. Therefore, assumptions on the ligand density need to be made. From the larger steric footprint of R5S as compared to that of HDT, it is expected that each R5S molecules replaces more than 1 HDT ligand. On the other hand, as HDT adopts a disordered conformation within the shell at the investigated temperatures, it exerts greater terminal steric hindrance than R5S, suggesting that R5S can be packed denser on the gold surface. Based on these considerations, we estimate that between 1 and 2 HDT molecules are replaced by each R5S molecule.

By extending eq. E21, the mass loss during the TGA experiment is given by

$$m_{\mathrm{loss}} = \frac{A_{\mathrm{AuNP}} \cdot [a \cdot \rho_{\mathrm{R5S}} + (1-a)\rho_{\mathrm{HDT}}] \cdot [a \cdot M_{\mathrm{R5S}} + (1-a) \cdot M_{\mathrm{HDT}}]}{N_{\mathrm{A}}}, \tag{E23}$$

with M_{R5S} the molar mass of R5S (88.17 g/mol) and a the number fraction of R5S within the shell. Rewriting yields an expression for a, given by

$$a = \frac{-A_{\mathrm{AuNP}} \cdot M_{\mathrm{HDT}} \cdot \rho_{\mathrm{R5S}} - A_{\mathrm{AuNP}} \cdot M_{\mathrm{R5S}} \cdot \rho_{\mathrm{HDT}} + 2 \cdot A_{\mathrm{AuNP}} \cdot M_{\mathrm{HDT}} \cdot \rho_{\mathrm{HDT}} - \sqrt{F}}{2 \cdot A_{\mathrm{AuNP}} \cdot M_{\mathrm{R5S}} \cdot \rho_{\mathrm{R5S}} - A_{\mathrm{AuNP}} \cdot M_{\mathrm{HDT}} \cdot \rho_{\mathrm{R5S}} - A_{\mathrm{AuNP}} \cdot M_{\mathrm{R5S}} \cdot \rho_{\mathrm{HDT}} + A_{\mathrm{AuNP}} \cdot M_{\mathrm{HDT}} \cdot \rho_{\mathrm{HDT}}} \tag{E24}$$

here F is defined as:

$$
\begin{aligned}
F = [A_{\mathrm{AuNP}} \cdot M_{\mathrm{R5S}} \cdot \rho_{\mathrm{HDT}}]^2 - 4 \cdot m_{\mathrm{loss}} \cdot N_{\mathrm{A}} \cdot A_{\mathrm{AuNP}} \cdot M_{\mathrm{R5S}} \cdot \rho_{\mathrm{HDT}} \\
+4 \cdot m_{\mathrm{loss}} \cdot N_{\mathrm{A}} \cdot A_{\mathrm{AuNP}} \cdot M_{\mathrm{HDT}} \cdot \rho_{\mathrm{HDT}} \\
-2 \cdot A_{\mathrm{AuNP}}^2 \cdot M_{\mathrm{HDT}} \cdot M_{\mathrm{R5S}} \cdot \rho_{\mathrm{HDT}} \cdot \rho_{\mathrm{R5S}} + [A_{\mathrm{AuNP}} \cdot M_{\mathrm{HDT}} \cdot \rho_{\mathrm{R5S}}]^2 \\
+4 \cdot m_{\mathrm{loss}} \cdot N_{\mathrm{A}} \cdot A_{\mathrm{AuNP}} \cdot M_{\mathrm{R5S}} \cdot \rho_{\mathrm{R5S}} \\
-4 \cdot m_{\mathrm{loss}} \cdot N_{\mathrm{A}} \cdot A_{\mathrm{AuNP}} \cdot M_{\mathrm{HDT}} \cdot \rho_{\mathrm{R5S}}
\end{aligned} \tag{25}
$$

The shell density ρ_{R5S} was selected based on the R5S-HDT exchange ratio. For a 1:1 exchange, $\rho_{\mathrm{R5S}} = \rho_{\mathrm{HDT}}$. For a 2:1 exchange, $\rho_{\mathrm{R5S}} = \frac{\rho_{\mathrm{HDT}}}{2}$.

Table S4 summarizes the results of all TGA experiments with additive-containing dispersions and their analyses using equations E17 to E25.

Table S4: Result of the TGA measurement of the R5S containing system

Parameter	AuNP@HDT	+ R5S 128 mM (1 d, 25 °C)	+ R5S 128 mM (5 h, 50 °C)
r_{core}	2.1 nm		
m_{gold}	14.03	17.47 mg	12.94 mg
m_{loss}	2.99	3.21 mg	1.27 mg
A_{total}	$1.037 \cdot 10^{18}$ nm^2	$1.29 \cdot 10^{18}$ nm^2	$9.57 \cdot 10^{17}$ nm^2
ρ_{LS}	6.71 nm^{-2}		
a	0	16.5% ± 4.5%	68.5% ± 13.5%

Thermal treatment of additive-free, R9S and R5N reference systems

AuNP coarsening in the presence of additive molecules is investigated by SAXS during heating from 40 °C to 100 °C in steps of 2 K. Figure S8 shows the obtained SAXS patterns of an additive-free AuNP dispersion (Figure S8a), and in the presence of R5S, R9S and R5N (Figure S8b, c, d, respectively).

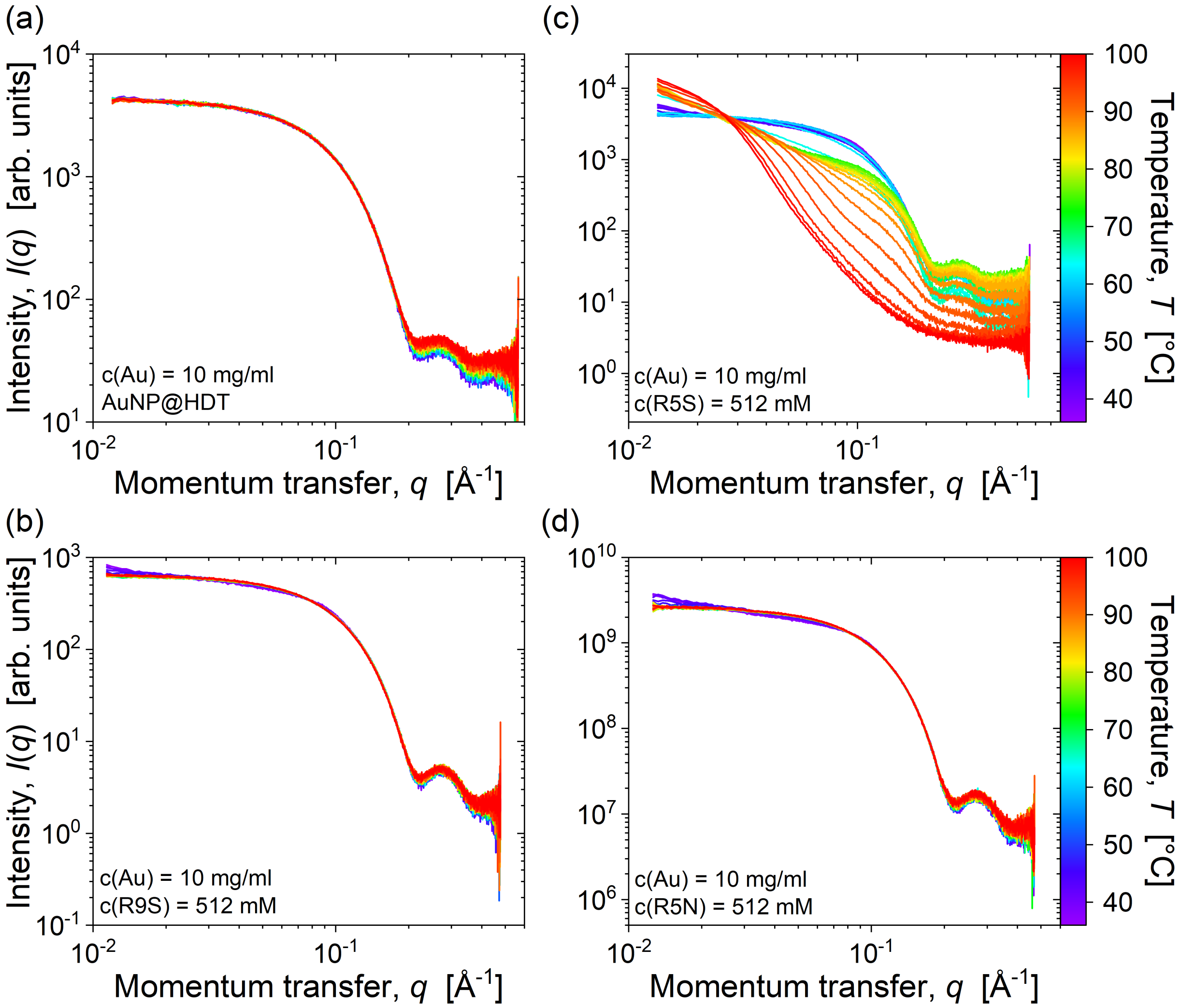

Figure S8: Temperature-dependent scattering curve of AuNP dispersions with a gold concentration of 10 mg/mL without additives (a), with R5S at a concentration of 512 mM (b), with R9S at a concentration of 512 mM (c), and with R5N at a concentration of 512 mM (d).

Small-angle neutron scattering (SANS)

Following the method described in our previous work,[8] the obtained SANS patterns on AuNP dispersions were modeled using the expression

$$I_{\mathrm{tot}}(q) = I_{\mathrm{pCS}}(q) + I_{\mathrm{OZ}}(q) + I_{\mathrm{Por}}(q) + I_{\mathrm{bkg}} \tag{E26}$$

with $I_{\mathrm{pCS}}(q)$ a form factor of polydisperse spherical core-shell particles, $I_{\mathrm{OZ}}(q)$ an Ornstein-Zernike structure factor, $I_{\mathrm{Porod}}(q)$ a Porod term (eq. E7), and I_{bkg} the incoherent background scattering.

$I_{\mathrm{pCS}}(q)$ is given by

$$I_{\mathrm{pCS}}(q) = \int_0^\infty G(r_{\mathrm{c}}) I_{\mathrm{CS}}(q, r_{\mathrm{c}}) dr_{\mathrm{c}} \tag{E27}$$

with r_{c} the core size, $I_{\mathrm{CS}}(q, r_{\mathrm{c}})$ the form factor of monodisperse core-shell particles and $G(r_{\mathrm{c}})$ the core radius distribution function, approximated by a Schultz-Zimm distribution. $I_{\mathrm{CS}}(q)$ is calculates as

$$I_{\mathrm{CS}}(q, r_{\mathrm{c}}) = N \left[\frac{3V_{\mathrm{c}}(SLD_{\mathrm{c}} - SLD_{\mathrm{s}}) J(qr_{\mathrm{c}})}{qr_{\mathrm{c}}} + \frac{3V_{\mathrm{c+s}}(SLD_{\mathrm{s}} - SLD_{\mathrm{m}}) J(q(r_{\mathrm{c}} + t_{\mathrm{s}}))}{q(r_{\mathrm{c}} + t_{\mathrm{s}})} \right]^2 \tag{E28}$$

Here is N the number density of the particles in the system, V_{c} the volume of the core, $V_{\mathrm{c\,+\,s}}$ the total particle volume, r_{c} the radius of the core, t_{s} the thickness of the shell, SLD_{c} the scattering length density of the core, SLD_{s} the scattering length density of the shell, SLD_{m} the scattering length density of the medium. $J(x)$ is given by

$$J(x) = \frac{\sin x - x \cos x}{x^2} \tag{E29}$$

Lastly, $I_{\mathrm{OZ}}(q)$ is given by

$$I_{\mathrm{OZ}}(q) = \frac{I_0}{1 + (q\xi_{\mathrm{OZ}})^2} \tag{E30}$$

with I_0 the scattering amplitude and ξ_{OZ} the correlation length of concentration fluctuations.

Figure S9 exemplarily shows a model fit according to eq. E26 including its individual contributions. In Figure S10, SANS patterns of AuNP dispersions in the presence of R5N and R9S are shown in Figure S10.

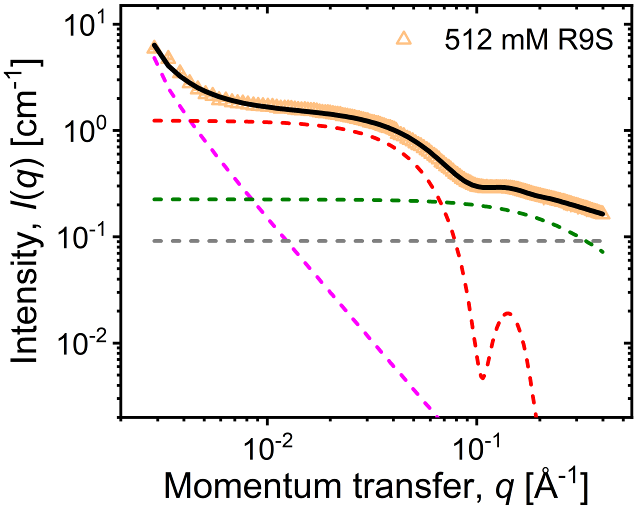

Figure S9: SANS pattern of 4 nm AuNPs dispersed in n-decane-d$_{22}$ at a concentration of 10 mg ml^{-1} in the presence of R9S at a concentration of 512 mM. Furthermore shown are a model fit according to eq. E26 (black line) and its individual contributions: $I_{\mathrm{pCS}}(q)$ (red dotted line), $I_{\mathrm{OZ}}(q)$ (green dotted line), $I_{\mathrm{Porod}}(q)$ (purple dotted line) and I_{bkg} (grey dotted line).

Diffusion-limited aggregation theory

The Smoluchowski coagulation equation is a differential equation that describes the time-evolution of particle number densities as they coagulate,[14] and is given by

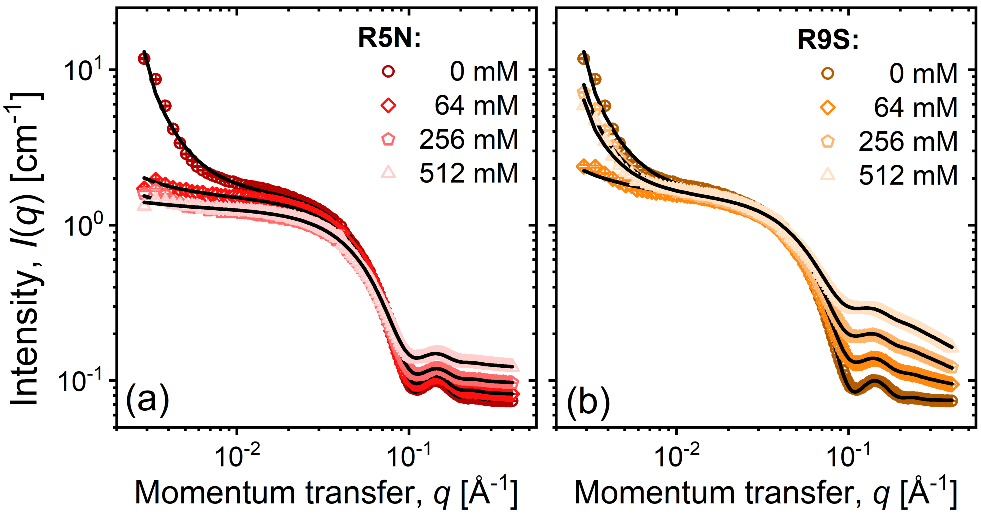

Figure S10: SANS patterns of 4 nm AuNPs dispersed in n-decane-d$_{22}$ at a concentration of 10 mg ml^{-1} in the presence of R5N (a) and R9S (b) at concentrations as indicated in the graphs. Black lines represent model fits according to eq. E26.

$$\frac{dn_j}{dt} = \frac{1}{2} \sum_{i+k=j} K_{ik}\, n_i\, n_k - n_j \sum_{i \geq 1} K_{ij}\, n_j \tag{E31}$$

with n_j the number concentration of particle clusters containing j primary particles, and K_{ij} a coagulation kernel, describing the rate of collisions between clusters containing i and j primary particles. K may assumed to be constant for agglomeration processes of narrowly-dispersed particles in their early stage.[15]

Following previous work,[15] the Smoluchowski coagulation equation can be solved analytically assuming a constant K, yielding the number concentration of primary particles as a function of time, $N(t)$, and is given by

$$N(t) = N_0 \frac{1}{1 + \frac{1}{2} K N_0 t} \tag{E32}$$

with N_0 the initial number concentration of primary particles.

Rearranging eq. E32 yields the time required to decrease the number of primary particles to a given fraction, $x = N(t_\mathrm{x})/N_0$, given by

$$t_x = 2\frac{1-x}{xKN_0} \tag{E33}$$

For diffusion-limited aggregation, K may be approximated by[15]

$$K = \frac{8k_\mathrm{B}T}{3\eta} \tag{E34}$$

with T the temperature, k_B the Boltzmann constant and η the dynamic viscosity of the solvent. Combining eqs. E33 and E34 gives

$$t_x \propto \frac{1}{K} \propto \frac{\eta}{k_\mathrm{B}T} \tag{E35}$$

Thus, the time required to decrease the number concentration of primary particles to a given value depends only on T and η.

References

(1) Knapp, T. V.; Dodange, S.; Monego, D.; Briones, C. M.; Hero, D.; Niebuur, B.-J.; Gallei, M.; Kraus, T.; Widmer-Cooper, A. Maintaining Shell Disorder with Kinked or Branched Ligands Stabilizes Apolar Nanoparticles. *ACS Nano* **2025**, *19*, 35127–35140.

(2) Cox, E. P. A Method of Assigning Numerical and Percentage Values to the Degree of Roundness of Sand Grains. *J. Paleontol.* **1927**, *1*, 179–183.

(3) Chen, F.; Johnston, R. L. Charge transfer driven surface segregation of gold atoms in 13-atom Au–Ag nanoalloys and its relevance to their structural, optical and electronic properties. *Acta Mater.* **2008**, *56*, 2374–2380.

(4) Gutiérrez-Wing, C.; Olmos-Asar, J.; Esparza, R.; Mariscal, M.; Yacamán, M. The role of ad-atoms in the coalescence of alkanethiol-passivated gold nanoparticles. *Electrochim. Acta* **2013**, *101*, 301–307.

(5) Azcárate, J. C.; Fonticelli, M. H.; Zelaya, E. Radiation Damage Mechanisms of Monolayer-Protected Nanoparticles via TEM Analysis. *J. Phys. Chem. C* **2017**, *121*, 26108–26116.

(6) Bo, A.; Kraus, T.; de Jonge, N. Temperature-Dependent Coalescence of Individual Nonpolar Gold Nanoparticles in Liquid. *ACS Appl. Nano Mater.* **2023**, *6*, 1146–1152.

(7) Pusey, P. In *Neutrons, X-rays and Light: Scattering Methods Applied to Soft Condensed Matter*; Lindner, P., Zemb, T., Eds.; North-Holland, 2002; Chapter Introduction to Scattering Experiments.

(8) Hasan, M. R.; Niebuur, B.-J.; Siebrecht, M.; Kuttich, B.; Schweins, R.; Widmer-Cooper, A.; Kraus, T. The Colloidal Stability of Apolar Nanoparticles in Solvent Mixtures. *ACS Nano* **2023**, *17*, 9302–9312.

(9) Porod, G. Die Röntgenkleinwinkelstreuung von dichtgepackten kolloiden Systemen. *Kolloid-Zeitschrift* **1951**, *124*, 83–114.

(10) Beaucage, G. Small-Angle Scattering from Polymeric Mass Fractals of Arbitrary Mass-Fractal Dimension. *J. Appl. Crystallogr.* **1996**, *29*, 134–146.

(11) Percus, J. K.; Yevick, G. J. Analysis of Classical Statistical Mechanics by Means of Collective Coordinates. *Phys. Rev.* **1958**, *110*, 1–13.

(12) Kaya, H. Scattering from cylinders with globular end-caps. *J. Appl. Crystallogr.* **2004**, *37*, 223–230.

(13) Knapp, T. V.; Hasan, M. R.; Niebuur, B.-J.; Widmer-Cooper, A.; Kraus, T. Stabilization of Apolar Nanoparticle Dispersions by Molecular Additives. *Langmuir* **2024**, *40*, 13527–13537.

(14) Smoluchowski, M. V. V. Mathematical Theory of the Kinetics of the Coagulation of Colloidal Solutions. *Z. Phys. Chem.* **1917**, *92*.

(15) Grant, S. B.; Kim, J. H.; Poor, C. Kinetic Theories for the Coagulation and Sedimentation of Particles. *J. Colloid Interface Sci.* **2001**, *238*, 238–250.
